# Production of Ochratoxin A and Citrinin and the Expression of Their Biosynthetic Genes from *Penicillium verrucosum* in Liquid Culture

**DOI:** 10.1021/acsomega.4c00874

**Published:** 2024-04-23

**Authors:** Marc Sasseville, Hai D. T. Nguyen, Simon Drouin, Adilah Bahadoor

**Affiliations:** † Applied Genomics, Human Health Therapeutics, National Research Council, 6100 Royalmount Ave, Montreal, Quebec H4P 2R2, Canada; ‡ Ottawa Research and Development Centre, Agriculture and Agri-Food Canada, 960 Carling Ave, Ottawa, Ontario K1A 0C6, Canada; § Metrology, National Research Council, 1200 Montreal Road, Ottawa, Ontario K1A 0R6, Canada

## Abstract

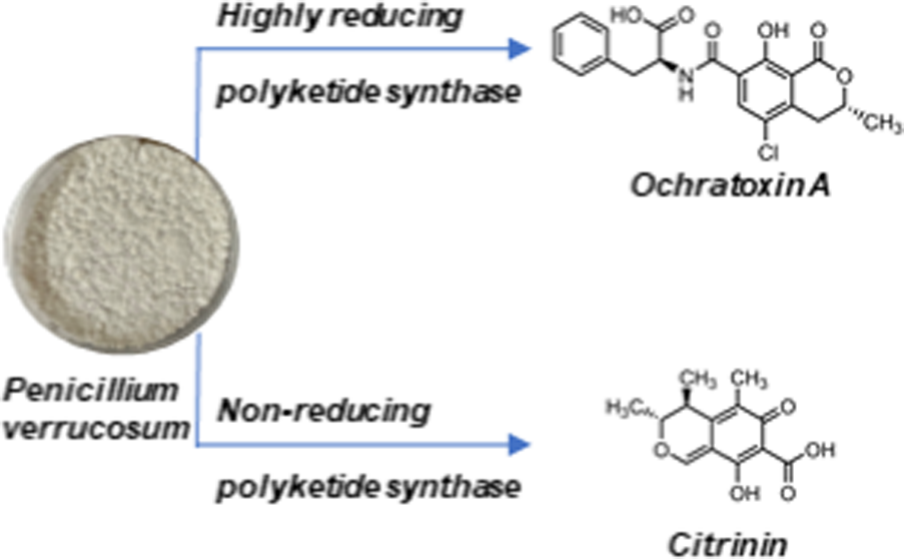

*Penicillium verrucosum* is a fungal pathogen capable of producing two mycotoxins of concern, ochratoxin A (OTA) and citrinin (CIT). The production profile of these two mycotoxins is not well understood but could help mitigate co-contamination in the food supply. As such, the production of OTA and CIT from *P. verrucosum* DAOMC 242724 was investigated under different growing conditions in liquid culture. We found that among the different liquid media chosen, liquid YES (yeast extract sucrose) medium induced the highest production of both OTA and CIT, when *P. verrucosum* DAOMC 242724 was cultured in stationary mode. Shake culture significantly reduced the amounts of OTA and CIT produced. Among all culture conditions tested, far greater amounts of CIT were produced compared to OTA. Consequently, upon transcriptomic data analysis, a statistically significant increase in the expression of CIT biosynthetic genes was easier to detect than the expression of OTA biosynthetic genes. Our study also revealed that the putative biosynthetic gene clusters of OTA and CIT in *P. verrusocum* DAOMC 242724 are likely distinct from each other. It appears that despite sharing a highly similar structure, the isocoumarin rings of OTA and CIT are each assembled by a specialized polyketide synthase enzyme. Our data identified a putative nonreducing polyketide synthase responsible for assembling the carbo-skeleton of CIT. In contrast, a highly reducing polyketide synthase appears to be involved in the biosynthesis of OTA.

## Introduction

**Figure 1:**
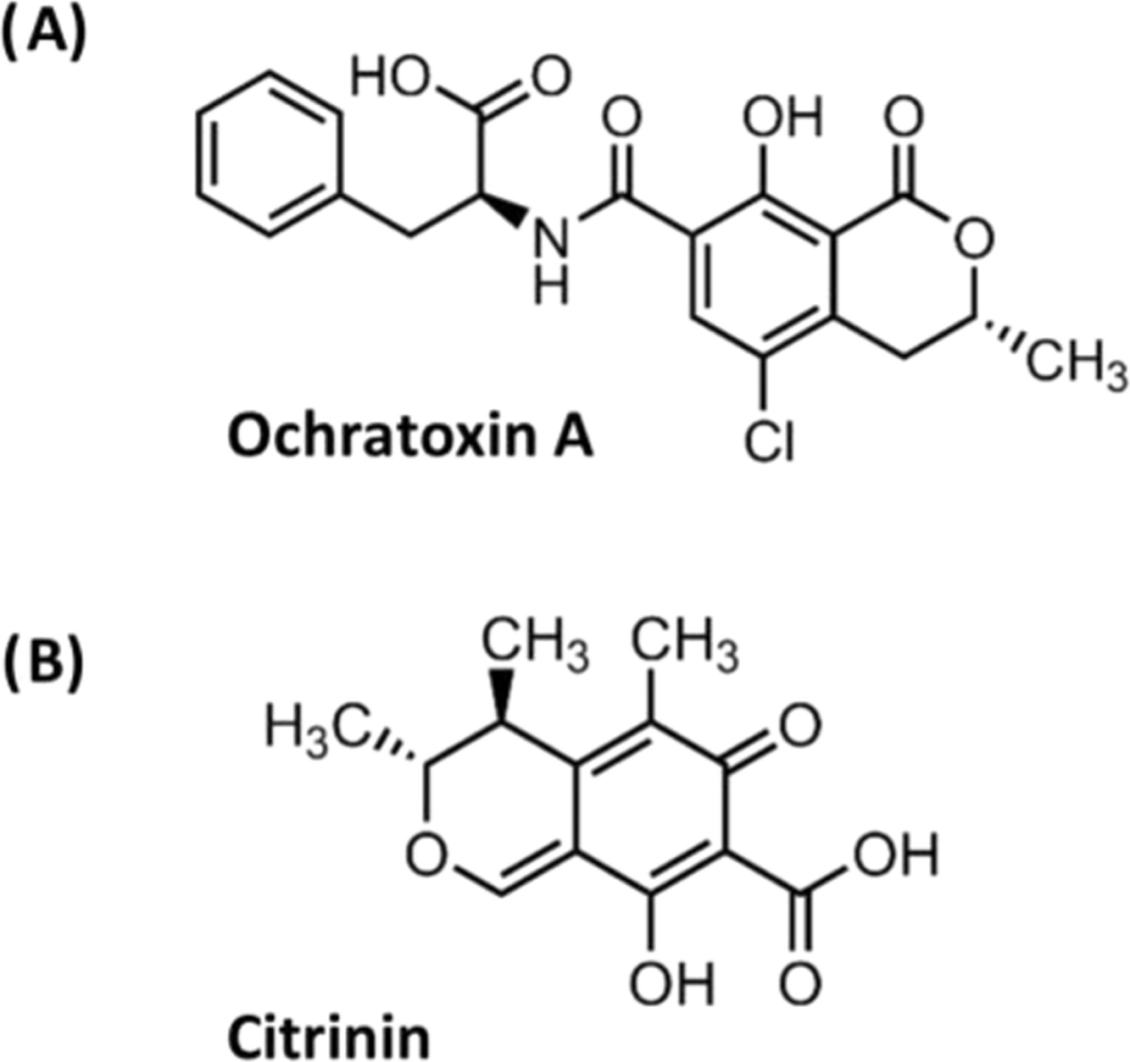
Structures of (A) ochratoxin A and (B) citrinin.

*Penicillium verrucosum* is a unique fungal crop pathogen capable of producing both the mycotoxins ochratoxin A (OTA) and citrinin (CIT) (Figure 1). Bucking the trend of OTA-only-producing *Aspergillus* and *Penicillium* strains or CIT-only-producing *Penicillium* and *Monascus* strains, the strains of *P. verrucosum* are the only ones identified to date as having the ability to produce both compounds.^1^

*P. verrucosum* thrives at colder temperatures such that it is uniquely positioned to threaten the supply of cereal grains coming from countries with a temperate climate. Major suppliers of cereals worldwide, such as Canada and Northern European countries, therefore have to maintain high vigilance in monitoring OTA and CIT contamination. While cereal crops are *P. verrucosum*’s natural hosts in the environment, the production of OTA and CIT typically occurs during the storage period of harvested grains, rather than in the field.^2,3^ Hence, the co-occurrence of OTA and CIT on cereal grain during storage is a serious concern as both are nephrotoxic agents,^4−6^ with OTA also classified as a probable carcinogen by the IARC.^7^ The serious adverse health side effects of consuming OTA- and CIT-contaminated grains have prompted global regulations to limit their concentrations in foodstuffs. As OTA is considered more toxic than CIT, several countries have a recommended maximum allowable level of OTA at 5 μg/kg in cereal commodities.^8^ Regulatory limits for CIT have so far been applied to fermented rice products at 2000 μg/kg.^9^

While the adverse health and economic impacts of *P. verrucosum* are clear, the genomic underpinnings of the biosynthesis of OTA and CIT are less well understood. As there are striking similarities between the OTA and CIT isocoumarin-like ring structures (Figure 1), it has been suggested that *P. verrucosum* could possess two similar sets of biosynthetic genes that are involved in producing OTA and CIT.^10^ The complete biosynthetic gene clusters directly implicated in the production of CIT and OTA have been identified from either CIT-only- or OTA-only-producing fungal species, respectively.^11,12^ The biosynthetic gene clusters from dual CIT- and OTA-producing *P. verrucosum* have not been identified to date. Using bioinformatic analyses, we identified putative OTA and CIT biosynthetic gene clusters of *P. verrucosum* DAOMC 242724. Our data suggest that the biosynthetic gene clusters of OTA and CIT from *P. verrucosum* are similar to their counterparts from OTA-only-producing *Penicillium nordicum* and CIT-only-producing *Monascus purpureus*, respectively. More importantly, the putative polyketide synthase (PKS) genes involved in OTA and CIT biosynthesis in *P. verrucosum* were found to belong to two different classes of PKS enzymes. According to Conserved Domain analysis,^13,14^ the predicted CIT-PKS was identified as a nonreducing PKS,^12,15^ in line with prior reports. On the other hand, the OTA-PKS was classified as a highly reducing PKS, suggesting that OTA and CIT are produced via two distinct biosynthetic pathways.

## Materials and Methods

### Preparation of Liquid Culture Media

*P. verrucosum* DAOMC 242724 was grown in either yeast extract sucrose (YES) medium with and without ammonium chloride supplementation, peptone yeast malt glucose (PYMG), or peptone yeast malt sucrose (PYMS) media. The following ingredients were dissolved in 1 L of water to prepare the YES medium: 30 g of yeast extract and 150 g of sucrose. Ammonium chloride (4 g/L) was added to supplement the YES medium with an inorganic source of nitrogen when required. To prepare PYMG medium, the following ingredients were dissolved in 1 L of water: 20 g of glucose, 3 g of NH_4_Cl, 2 g of KH_2_PO_4_, 2 g of MgSO_4_·7H_2_O, 0.2 g of FeSO_4_·7H_2_O, 2 g of yeast extract (Difco or Aldrich), 2 g of malt extract (Difco or Aldrich), and 2 g of peptone (Difco or Aldrich). The preparation of PYMS medium was similar to that of PYMG medium, except glucose was substituted with 20 g/L sucrose. The liquid culture media were sterilized by filtration (Millipore, 0.22 μm) prior to use. YES medium was sterilized by autoclave when *P. verrucosum* DAOMC 242724 was grown in shake culture only.

### Fermentation of *P. verrucosum* DAOMC 242074 in YES Medium Shake Culture

An agar slant of *P. verrucosum* DAOMC 242724 growing on 2% Blakeslee’s malt extract agar (MEA) was macerated in sterile distilled deionized H_2_O and a 5% (v/v) aliquot was used to inoculate ten 250 mL Erlenmeyer flasks containing 50 mL of the autoclaved PYMG media. Flasks were incubated on a rotary shaker (100 rpm) at 25 °C for 5 days. After the initial incubation period, starter cultures were combined, macerated, and subsequently used to inoculate 40 second-stage cultures in 250 mL Erlenmeyer flasks containing 100 mL of yeast extract sucrose. Second-stage cultures were incubated as previously described where six flasks of each medium were removed at 24 h intervals for a total of 144 h.

### RNA Extraction and Sequencing

Second-stage cultures were individually filtered through a Whatman #4 (Whatman GE Healthcare, U.K.) by suction to separate the mycelia from the filtrate. Mycelia from three flasks at each time point were rinsed with sterile distilled deionized H_2_O, placed in centrifuge tubes, flash-frozen with liquid nitrogen for 1 min, and stored at −80 °C prior to RNA extraction. To capture the transcriptome of *P. verrucosum* DAOMC 242724 while synthesizing OTA, RNA was extracted from cultures incubated in YES broth with the Nucleospin II RNA kit (Macherey-Nagel) following the manufacturer’s instructions. Sequencing of RNA libraries (101 bp paired-end) was performed on an Illumina HiSeq 2500 with TrueSeq V3 chemistry at the National Research Council Canada in Saskatoon, Saskatchewan, Canada.

### Quality Control of Reads

The quality of the RNA-Seq reads was assessed using FastQC v0.10.1 (http://www.bioinformatics.babraham.ac.uk/projects/fastqc/). Low-quality bases and contaminating adaptor sequences were removed with Trimmomatic v0.35.^16^ Error correction was performed on the trimmed reads with BayesHammer.^17^

### Transcriptomic Analysis of *P. verrucosum* DAOMC 242724 as It Produced OTA and CIT

The first step in this study was to build a transcriptome to enable downstream gene identifications and perform differential gene analyses. To this end, RNA-Seq data from NCBI BioProject PRJNA292129 was used, it derives from RNA harvested in triplicates from Day 1 to Day 6, as *P. verrucosum* DAOMC 242724 grew in second-stage YES medium, to generate a time-dependent RNA library (Supporting Information Table S1). Trinity-v2.12^18^ software was used to perform *de novo* transcriptome assembly from 10 RNA-Seq samples (SRR2168737, SRR2168738, SRR2167777, SRR2167776, SRR2168735, SRR2148672, SRR2148671, SRR2148670, SRR2148669, SRR2148662) out of 18 from the data set, using about 200 M reads from different time points. Reducing the number of RNA-Seq samples to 10 was necessary to limit analysis time and computational resources. To compensate for the shortcomings of using unstranded RNA sequences^19^ with Trinity and the prospect of obtaining fused genes because of the close proximity of genes on the *Penicillium* genome, all open reading frames (ORFs) from both sense and antisense frames were identified from the Trinity transcripts.

To select appropriate stranded coding sequences (CDS) from the Trinity assembled transcripts, we aligned them and all coding transcripts (229 663) from a total of 686 *Penicillium* genomes in the European Nucleotide Archive (ENA) to the genome of *P. verrucosum* BFE808 (accession no. LAKW00000000.2) (Supporting Information Table S2) using gmap version (2021–07–23).^20^ After the alignments were performed, AUGUSTUS^21^ tool from OmicsBox/BLAST2GO was used to predict CDS on the *P. verrucosum* BFE808 genome assembly.

The best Trinity assembled transcripts of *P. verrucosum* DAOMC 242724 were selected using an in-house R script, keeping the longest ORFs that overlapped with either the European Nucleotide Archive (ENA)-matching transcripts or the AUGUSTUS-generated CDS. Newly identified spliced genes, without overlap, were also kept, discarding any unspliced genes unlikely to be true genes, as they did not overlap with any known transcript. A preliminary list of 12 983 genes was obtained at the end of this process.

### OTA and CIT Biosynthetic Gene Cluster Identification

The following GenBank gene sequences were aligned using BLAST^22,23^ against the CDS sequences of the *P. verrucosum* DAOMC 242724 transcriptome to identify the genes for CIT biosynthesis: *Marmoricola aurantiacus* citrinin biosynthesis gene cluster (accession no: EU309474.1), *Monascus ruberpksCT* gene sequence (accession no: AB167465.1), *M. ruber* citrinin biosynthesis gene cluster (accession no: KT781075.1), and *M. purpureus* (accession no: AB243687.1). For OTA, the biosynthetic gene cluster was identified from *P. nordicum* DAOMC 185683 (accession no: MG701895.1) was used. Once CIT and OTA biosynthetic gene sequences were identified, the function of the enzymes that they encoded was predicted using Conserved Domain.^13,14,24^

### Differential Gene Level Expression Analysis

The newly identified genes coordinate serves to produce a gene count matrix from RNA sequencing samples using Salmon v1.4.0.^25^ Gene level (12,983 genes) differential expression was done using the Deseq2 v1.30.0 Bioconductor R package^26^ comparing days 2–6 against day 1.

### OTA and CIT Quantitation from *P. verrucosum* DAOMC 242074 Grown in YES Medium under Shake Culture

Three flasks were randomly selected for harvest on days 1, 2, 3, 4, 5, and 6. The harvested liquid culture was sterilized by filtration at 0.22 μm and stored at −80 °C until quantitation. For OTA quantitation, the media harvested on days 4, 5, and 6 were diluted 10-fold by mixing 200 μL of the medium with 1.8 mL of 50% acetonitrile in water +0.1% formic acid, containing a known amount of the OTAL-1 solution^27^ (a ^13^C_6_–OTA certified reference material). Liquid media samples harvested on days 1, 2, and 3 were diluted 1:1 in the same solvent as described above. As no appropriately labeled internal calibrant was available to quantitate CIT, single-point standard addition was used instead. The culture samples were diluted 10-fold as described above. A 150 μL aliquot of each diluted sample was transferred to a vial insert served as the unknown sample. A second 150 μL aliquot was treated with 50 μL of a CIT standard solution at a concentration of (0.029 μg/mL) served as the calibrant. The samples were analyzed by high-resolution LC-MS on a Vanquish UHPLC unit coupled to an Exploris mass spectrometer, both from Thermo Fisher Instruments. Samples were cooled to 8 °C in the autosampler during the sequence run and each was analyzed at an injection volume of 2 μL. High-resolution mass spectral data were recorded in positive mode at a resolution of 60 000, an RF value of 70%, and a scan range of *m*/*z* = 100–1000 amu. Tuning parameters for the heated electrospray ionization (H-ESI) source were as follows: spray voltage 3.5 kV, sheath gas flow 30 units, auxiliary gas flow 10 units, sweep gas flow 2 units, ion transfer tube temperature 350 °C, and a vaporizer temperature of 250 °C. High-resolution mass spectral data were recorded in positive mode at a resolution of 60 000, RF value of 70%, and Elution by UHPLC proceeded at 0.25 mL/min on an ACE-C18-PFP column (C18, 50 × 2.1 mm^2^, 1.7 μm) heated to 40 °C, using a mobile phase consisting of acetonitrile:water modified 0.1% formic acid, and the following gradient: 0–4 min (1% acetonitrile), 4–14 min (1–90% acetonitrile), 14–18 min (90% acetonitrile), 18–19 min (90–1% acetonitrile) and 19–22 min (1% acetonitrile). According to these LCMS parameters, OTA eluted at 10.9 min and CIT eluted at 10.3 min (Supporting Information Figures S2 and S3). Both compounds were detected by extracted ion chromatogram (EIC) from their exact mass centered within a 5 ppm window and the subsequent peaks integrated using Xcalibur software. Area under curve (AUC) for each peak detected was used to calculate OTA concentrations by single-isotope dilution mass spectrometry according to the method described previously.^28^ CIT concentrations were obtained graphically using the equation of a line according to a standard addition protocol.

### Fermentation of *P. verrucosum* DAOMC 242074 in Stationary Cultures

*P. verrucosum* DAOMC 242724 was grown on Blakeslee’s malt extract agar at 20 °C. After 7 days, an approximately 1 cm square piece of agar was used to inoculate individual sterile 250 mL Erlenmeyer flasks each containing an autoclaved glass microfiber filter (Cytiva, Whatman 934-AH, 70 mm) and 50 mL of either peptone yeast malt sucrose (PYMS) or yeast extract sucrose (YES) supplemented with ammonium chloride. Four biological replicates for each media type were set up. The culture flasks were grown in stationary phase in the dark at 20 °C for 42 days. To sample the growing cultures during the time course, aliquots were aseptically removed on days 0, 5, 8, 10, 12, 14, 16, 20, 28, 35, and 42. Aliquots were diluted 10-fold at 200 μL in 1.8 mL of 50% acetonitrile in water +0.1% formic acid. Diluted samples were kept at −80 °C until ready for quantitation.

### Quantitation of OTA and CIT from *P. verrucosum* DAOMC 242074 in Stationary Cultures

The frozen 10-fold diluted samples were allowed to reach room temperature. To quantitate OTA, each sample was treated with 40 μL of OTAL-1, mixed by vortexing, centrifuged at 13 000*g* for 4 min, and then filtered at 0.22 μm via disposable PTFE disposable syringe filters before injecting on the LC-MS instrument. To prepare the samples for CIT quantitation in the absence of an isotopically labeled calibrant, single-point standard addition was performed. About 23 g of an in-house CIT standard solution was prepared at 1.31 μg/g in acetonitrile:water:formic acid (1:1:0.01) and filtered at 0.22 μm. A 50 μL aliquot of each of the 10× diluted solutions prepared above for OTA quantitation was further diluted in either 950 μL of the CIT standard solution to serve as the calibrant or in 950 μL of acetonitrile:water:formic acid (1:1:0.01) to serve as the sample for LC-MS quantitation. The samples for CIT quantitation were therefore diluted 200-fold. The samples were analyzed by high-resolution LC-MS on a Vanquish UHPLC unit coupled to an Exploris mass spectrometer, both from Thermo Fisher Instruments as described above for the shake cultures. Quantitation of OTA was obtained by single-isotope dilution mass spectrometry, while CIT measurements were obtained from single-point standard addition. Data analysis was performed by obtaining the area under curve (AUC) for each analyte by using the QuanBrowser application of Xcalibur Software 4.0. The data was exported to Excel for statistical evaluation and graphical representation. The OTA and CIT quantitation at each time point was an average of four biological replicates. The standard error was calculated as the ratio of the standard deviation to *n* – 1, where *n* = 4.

## Results

### Transcriptome of *P. verrucosum* DAOMC 242724

The next-generation sequence reads obtained were mapped using Salmon software onto the assembled transcriptome of *P. verrucosum* DAOMC 242724 achieving an overall mapping rate of 59% (Supporting Information Figure S1). Alignment of some RNA-Seq samples with the splice-aware tool STAR (Spliced Transcripts Alignment to a Reference) showed a mapping rate ranging between 75 and 91% (data not shown).^29^ The differences between the two alignment methods were deemed to be due to STAR taking into consideration splicing, noncoding RNA, and untranslated regions (UTR).

### OTA Biosynthetic Gene Cluster of *P. verrucosum* DAOMC 242724

Our aim was to find the putative OTA biosynthetic genes of *P. verrucosum* DAOMC 242724 using transcriptome data. Five candidate genes were found to match the genes in the previously described OTA biosynthetic gene cluster of *P. nordicum* DAOMC 185683.^30^ The *P. nordicum* cluster contains five main genes: *otaA* encoding for a polyketide synthase (PKS), *otaB* a nonribosomal peptide synthetase (NRPS), *otaC* a Cyp450 monooxygenase, *otaD* a halogenase, and *otaR1* a bZIP transcription factor. Using BLASTn, a comparison of the known OTA genes with candidate genes from *P. verrucosum* DAOMC 242724 revealed a high level of similarity (Table 1 and Supporting Information Table S3).^22^ In addition, the recently identified SnoaL cyclase encoding gene *otaY*(31) was also found in the transcriptomic data. A search for highly similar sequences to all six OTA genes found sequences with 93–100% similarity on Contig 1009 (LAKW02001000.1) of the whole genome sequence of *P. verrucosum* BFE808 (Supporting Information Table S4).

**Table 1:** Putative OTA Biosynthetic Genes from *P. verrucosum* DAOMC 242724

putative OTA genes in *P. verrucosum* DAOMC 242724	% identity with OTA genes from *P. nordicum* DAOMC 185683	function
g07677 (*otaD*)	98%	halogenase
g07678 (*otaR1*)	97%	bZIP transcription factor
g07680 (*otaC*)	98%	Cyp450
g07679 (*otaB*)	98%	NRPS
g09503 (*otaA*)	97%	PKS
g07681 (*otaY*)	99%	SnoaL cyclase

### CIT Biosynthetic Gene Clusters of *P. verrucosum* DAOMC 242724

A similar approach was used to identify the CIT biosynthetic cluster in *P. verrucosum* DAOMC 242724, where known CIT biosynthetic genes served as a template to identify their orthologs from *P. verrucosum* DAOMC 242724. Numerous genes comprise the CIT biosynthetic gene cluster, and these genes have been identified in *M. purpureus*, *M. aurantiacus*, and *M. ruber*. The most important gene encoding the PKS enzyme (also known as *CitS*), responsible for assembling the isocoumarin moiety of CIT, was fully characterized in *M. purpureus* (accession no: AB167465.1) and *M. ruber* (accession no: KT781075.1) (Table 2).^12,32^ Subsequently, other components of the biosynthetic cluster were identified in *M. purpureus* and *M. aurantiacus*.^11,33^ As shown in Table 2, six CIT biosynthetic genes belonging to *P. verrucosum* DAOMC 242724 were identified (Table 2 and Supporting Information Table S5). It should be noted that the gene sequence encoding the CIT-PKS was obtained as two separate gene sequences, g03741 and g03742, where the portion comprising g03742 was fused to another sequence of the library. However, it is very likely that this fused partial sequence is still only a part of the whole CIT-PKS transcript. Finally, whole genome analysis unveiled the location of the CIT genes on contig 10 (LAKW02000010.1) of the *P. verrucosum* BFE808 genome assembly (Supporting Information Table S6).

**Table 2:** Putative CIT Biosynthetic Genes from *P. verrucosum* DAOMC 242724

	% similarity with corresponding CIT genes from			
putative CIT genes in *P. verrucosum* DAOMC 242724	*M. purpureus*	*M. ruber*	*M. aurantiacus*	function
g06943		*citC* (82%)	*ctnD* (82%)	oxidoreductase
g06944	*ctnR (85%)*			transcriptional factor
g06947		*citE* (87%)	*ctnE* (87%)	short-chain dehydrogenase
g06948	*ctnB* (98%)	*citA* (85%)		serine hydrolase
g03740	*ctnC* (99%)			MFS transporter
g03741–42	*pksCT* (92%)	*citS* (87%)		PKS

### Production of OTA and Its Gene Expression in YES Medium in Shake Culture

**Figure 2:**
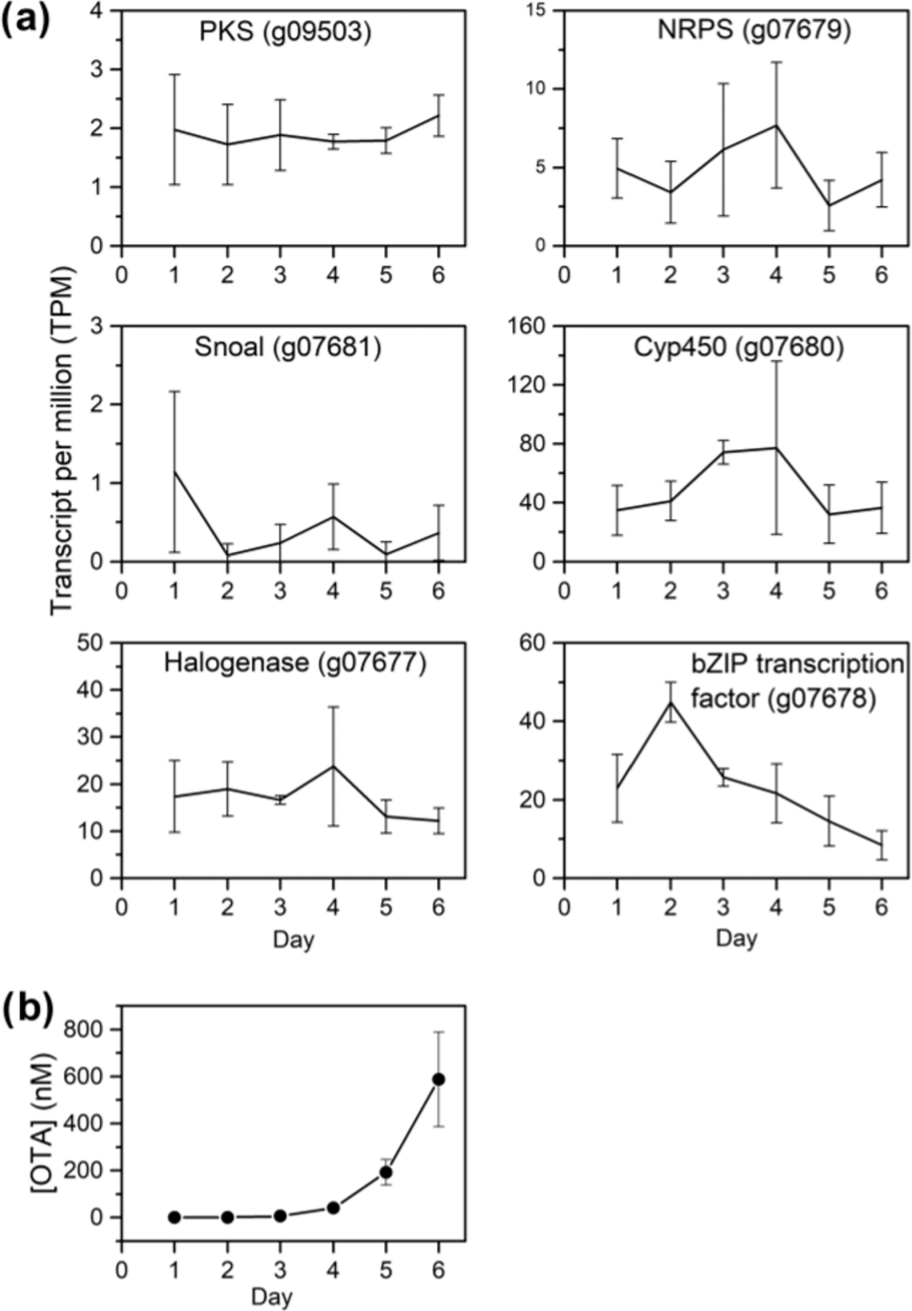
(A) OTA biosynthetic gene expression over 6 days in YES liquid medium under shake culture. (B) Concentration of OTA over 6 days of growth in YES medium.

When gene counts for the OTA biosynthetic genes were analyzed over the 6-day culture, no significant increase in their expression was discerned (Figure 2A). In contrast, the concentration of OTA steadily increased from day 3 to day 6, rising from approximately 6–587 nM (Figure 2B). On days 1 and 2, the amount of OTA quantified was negligible at <1 nM. The amount detected was more or less constant, likely indicative of minor OTA production that may have occurred in the first-stage medium and carried over in the second-stage YES medium.

The absolute quantitation of OTA was feasible due to the availability of a certified reference material (CRM) for ^13^C-isotopically labeled OTA, OTAL-1. With a certified concentration for ^13^C_6_–OTA, OTAL-1 served as both an internal standard and primary calibrant to quantify OTA present in YES medium.^27,28^ As a precaution, to ensure that large variabilities in the amounts of dilution solvent containing OTAL-1 used were not a factor influencing the quantitation of OTA, the intensities of ^13^C_6_–OTA from OTAL-1 were plotted for all three biological replicate samples and time points (Supporting Information Figure S4). The vast majority of the responses were within two standard deviations of the mean, underscoring that slight variations in the volume of OTAL-1 or fluctuating instrument sensitivity during the sequence did not have undue influence on the measurements of OTA.

### Production of CIT and Its Gene Expression in YES Medium in Shake Culture

**Figure 3:**
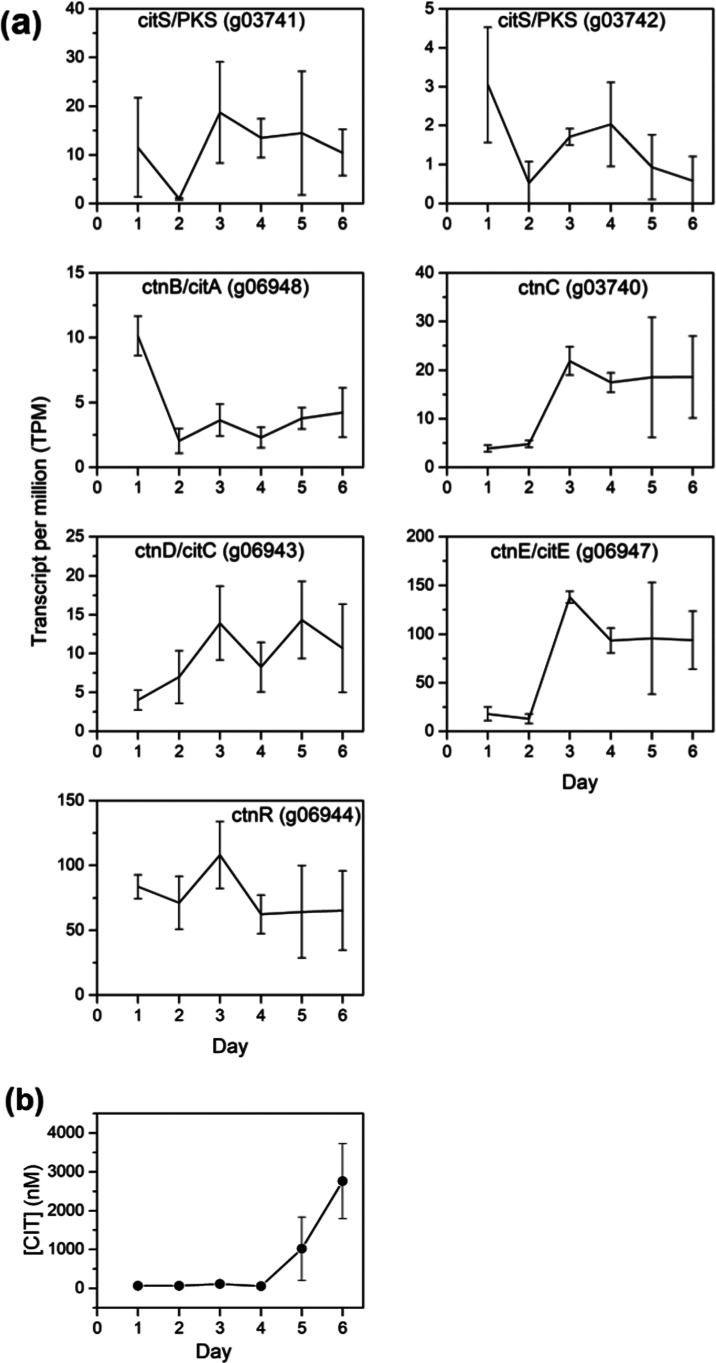
(a) CIT biosynthetic gene expression over 6 days grown in shake culture in YES medium. (b) Production of CIT over 6 days in YES medium.

CIT accumulated faster than OTA in the YES medium (Figure 3B). Similar to OTA, the amount of CIT remained relatively unchanged until Day 4 at about 66 nM. After this initial stage, CIT production accumulated to nearly 42-fold to reach 2763 nM by Day 6. The higher concentrations observed for CIT were echoed in observable increase in the expression of a number of the CIT biosynthetic genes, including tailoring genes *ctnD*/*citC* and *ctnE*/*citE* and the MFS transporter encoding gene *ctnC* (Figure 3A). Surprisingly, the anchor gene encoding the PKS, *citS*, did not show a statistically significant increase in gene counts.

Unlike OTA, CIT does not have a suitable isotopically labeled standard. Thus, to avoid incorrect measurements from matrix effects, CIT concentrations were obtained from single-point standard addition. As such, each biological replicate was analyzed as a pair, including the plain culture media samples from which CIT is to be quantified and a standard solution comprising the same liquid media to which a known amount of a standard solution of CIT was added. The concentration of CIT in the sample was obtained by plotting a straight line from the two samples.

### Production of OTA and CIT in Stationary Cultures

**Figure 4:**
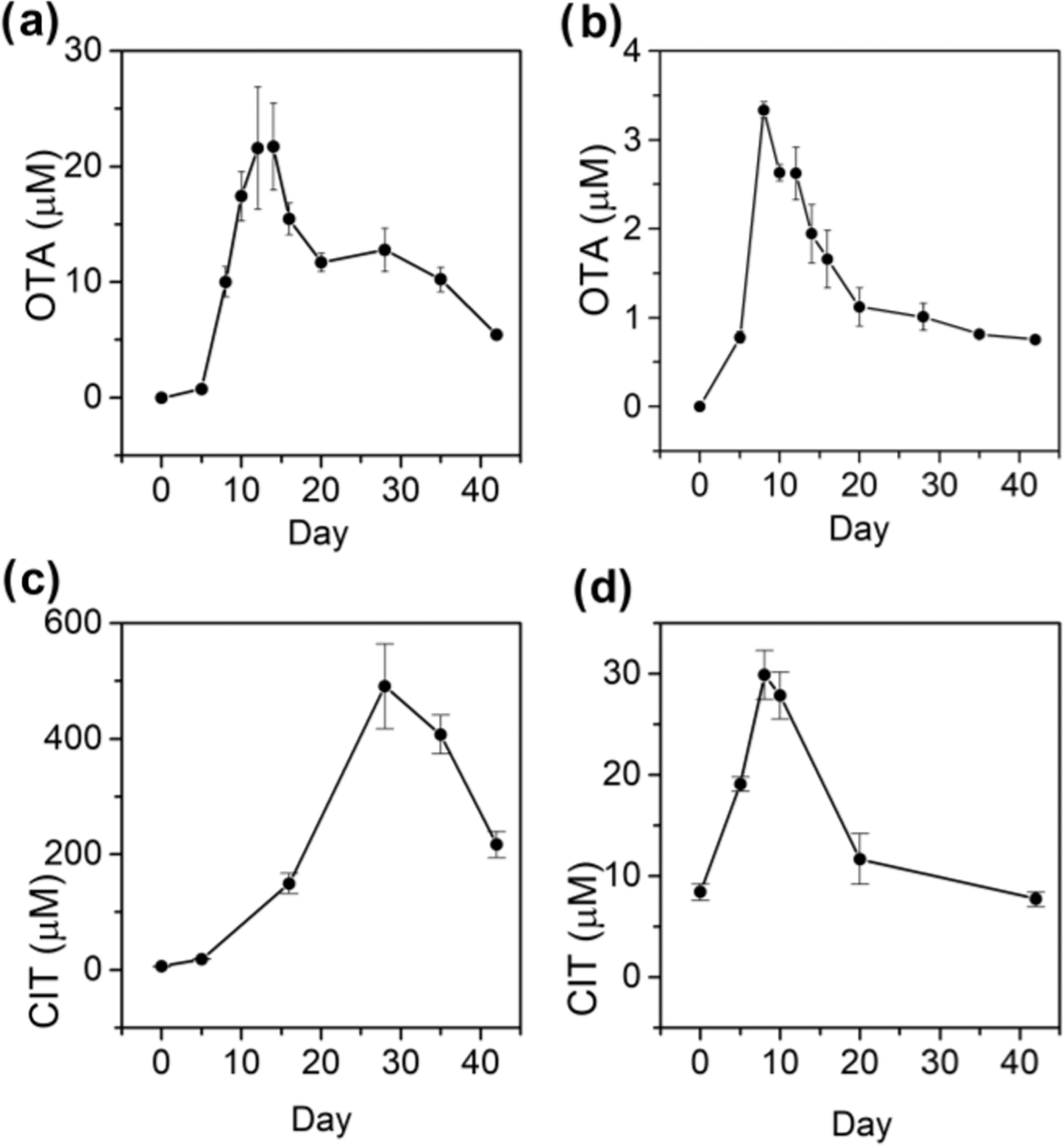
Production OTA and CIT in stationary cultures: (a) OTA quantitation in YES medium supplemented with ammonium chloride; (b) OTA quantitation in PYMS medium; (c) CIT quantitation in YES medium supplemented with ammonium chloride; (d) CIT quantitation in PYMS medium.

As shown above, the rapid increase in the production of OTA by days 5 and 6 in shake culture was not accompanied by an expected increase in the expression of its biosynthetic genes. Although the YES medium is generally a common growth medium for *P. verrucosum* to induce OTA production, it was possible that other factors were mitigating its production. Stationary cultures and different liquid media recipes were tested to see if these new conditions would be more conducive to the production of OTA. In stationary YES medium supplemented with ammonium chloride and in PYMS medium, significantly more OTA was secreted (Figure 4a,b). Unlike a peak OTA concentration of 587 nM obtained from the two-stage shake culture in YES medium described above, culturing *P. verrucosum* DAOMC 242724 in one-stage stationary YES medium supplemented with ammonium chloride significantly increased the amounts of OTA produced, peaking at a concentration of 22 μM, a nearly 37-fold increase. In PYMS medium, the effects were more subdued, but an increase in the concentration of OTA was still observed at a maximum of 3.3 μM. Under both stationary culture conditions, the peak concentrations of OTA were achieved later during the growth period, typically after 8 days of growth, compared to 6 days in two-stage shake culture. To determine the absolute concentrations of OTA, the certified reference material (CRM) for ^13^C_6_-isotopically labeled OTA, OTAL-1 was used.^28^ The intensity of ^13^C_6_–OTA throughout the sequence remained within two standard deviations of the mean, to confirm that the instrument drift and large variations in the volume of the CRM did not adversely affect the absolute quantitation in YES medium (Supporting Information Figure S5) or PYMS medium (Supporting Information Figure S6).

The stationary culture had a more pronounced effect on the production of CIT. In YES medium supplemented with ammonium chloride, peak production of CIT was observed on day 28 at 490 μM, a 175-fold increase compared to a maximum concentration of 2.8 μM in the two-stage shake culture in YES medium (Figure 4c). In stationary PYMS medium, CIT production peaked at 29 μM, still a 10-fold increase compared to 2.8 μM in a two-stage shake culture in YES medium (Figure 4d). Although the production profile of CIT in both media types was monitored on the same schedule as OTA, the absolute quantitation was performed on select days only to reduce the number of samples for LCMS analysis. The full profile of CIT production is shown here (Supporting Information Figures S7 and S8).

### PKS Enzyme in the OTA and CIT Biosynthetic Gene Clusters

**Figure 5:**
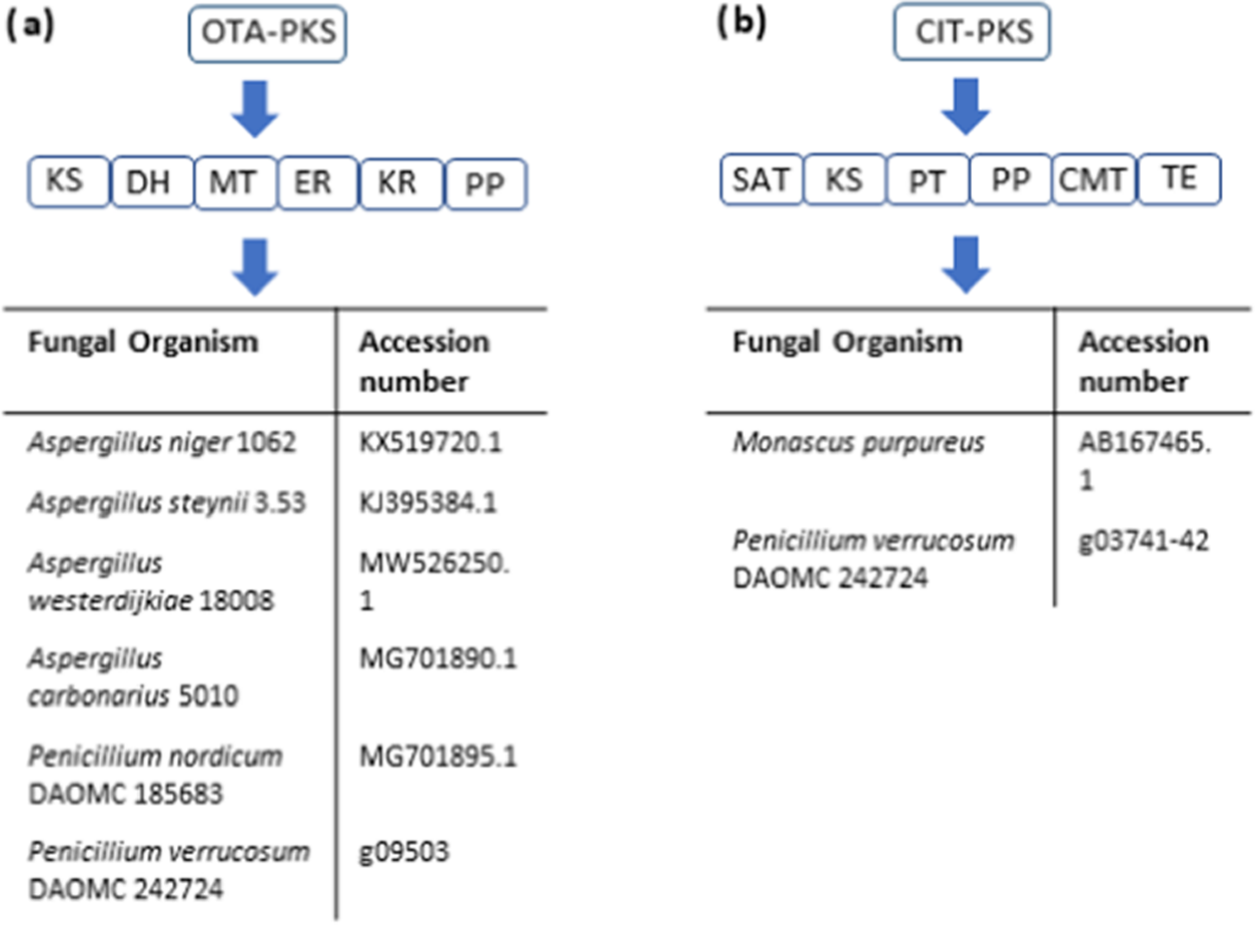
(a) Functional domains of the OTA polyketide synthase from OTA-producing *Aspergillus* and *Penicillium* species. (b) Domains of the CIT polyketide synthase from CIT-producing *M. purpureus* and *P. verrucosum* DAOMC 242724.

Since gene expression in shake culture did not provide statistically significant results for OTA, a bioinformatic analysis of the OTA-PKS enzyme was performed to determine its structure and function in greater detail. An analysis of the different domain arrangements of the OTA-PKS and CIT-PKS genes unveiled from *P. verrucosum* DAOMC 242724 was performed. Conserved Domain^13,14^ analysis of the protein sequence encoded by the OTA-PKS gene (g09503), revealed that the OTA-PKS as a highly reducing polyketide synthase containing six domains in the following arrangement: a ketoacyl synthase (KS), a dehydratase domain (DH), a methyl transferase domain (MT), an enoyl reductase domain (ER), a keto reductase domain (KR), and a phosphopantetheine arm (PP) (Figure 5).^13,14,24^ Similar domain arrangements were obtained for the OTA-PKS identified from *P. nordicum* DAOMC 185683 and several *Aspergillus* species (Figure 5a and Supporting Information Figure S9).

In contrast, the CIT-PKS (citS) has been characterized as a nonreducing polyketide synthase, possessing a starter acyl transferase unit (SAT), a KS domain, a product template (PT) domain, a PP arm, a C-methyl transferase domain (CMT), and a thioesterase domain (TE) for product release (Figure 5b and Supp. Info Figure S10).^12,15,32^ The corresponding sequence for the CIT-PKS identified from *P. verrucosum* DAOMC 242724 provided a similar domain arrangement, including the requisite PT domain and CMT domain, which Conserved Domain correctly classified as a S-adenosyl-methionine (SAM)-dependent methyl transferase as previously reported.^13−15^ The SAT and KS domains were missing, thus suggesting that the sequence identified for *citS* (g03741–42) is incomplete (Supporting Information Figure S11).

To obtain further proof that the OTA- and CIT-PKS enzymes are distinct, a BLASTn analysis was performed.^22,23^ A search for similar proteins showed that the OTA-PKS clustered among similar enzymes from *Penicillia* and *Aspergilli* with greater than 80% similarity, but none that were attributed to a CIT-only-producing organism (Supporting Information Table S7). The CIT-PKS was highly similar to its counterpart from *P. expansum* and several *Monascus* species, known to produce CIT but not OTA (Supporting Information Table S8).

## Discussion

The co-contamination of stored grains with OTA and CIT is well documented.^2,3^ As the more toxic metabolite, OTA contamination has been studied to a greater extent than CIT. Our efforts to study the simultaneous production of the two mycotoxins have unveiled that when *P. verrucosum* was grown in liquid medium, more CIT than OTA was produced. It was reported that under similar conditions, the production of CIT was favored if the *P. verrucosum* culture was subjected to oxidative stress induced by either addition of Cu^2+^ ions to the culture medium or light exposure during the growth stage.^34,35^ The data presented here, however, suggest that even in the absence of known stressors, CIT production would still be higher compared to OTA.

Our study also linked transcriptomic changes occurring in *P. verrucosum* as it grew in liquid YES medium under a shake culture. A clear change in the transcriptome was noted from Day 3, to coincide with the initial uptick in OTA and CIT production (Supporting Information Figure S12). A few subsets of genes exhibiting similar expression patterns were subsequently found to be upregulated or downregulated over the 6-day culture; however, determining the function of the genes with gene deletion experiments was beyond the scope of this study (Supporting Information Figures S13–S17). While it was clear that a group of genes were clearly upregulated as of Day 3, the OTA and CIT biosynthetic genes were not among them (Supporting Information Figures S15 and S16). A mitigated 2- to 6-fold increase in the expression of a few CIT biosynthetic genes was observed (Figure 3a), but no statistically relevant upregulation of OTA biosynthetic genes was discerned (Figure 2a).

Both OTA and CIT were quantified within the constraints of a six-day transcriptomic library. However, subsequent data collected in single-stage YES medium showed that peak production of OTA and CIT occurred on Day 11 and Day 28 respectively over a 42-day growth period (Figure 4). More significantly, the data showed that up to 37-fold more OTA and up to 175-fold more CIT were produced in stationary YES medium compared to the shake culture. Had the transcriptomic data been collected under such circumstances, the upregulation of the biosynthetic genes, especially those for OTA, could have been clearer. Our results suggest that prior to obtaining transcriptomic libraries, a good practice would be to screen a few different culture conditions to determine the best-suited growth conditions that elicit a maximum production of the desired metabolites. It would make sense that the higher the concentration of the metabolites, the higher the likelihood that the corresponding biosynthetic genes would be significantly upregulated, improving the chances of unambiguously identifying a suspected increase in gene expressions. However, in our study, the transcriptomic library was obtained from the two-stage shake culture in YES medium first, without exploring other culture conditions. Once it was determined that the gene counts did not necessarily mirror the accumulation profile of OTA and CIT in shake culture, alternate growth conditions were explored. Even though stationary growth in YES and PYMS medium were clearly superior at inducing OTA and CIT production (Figure 4), it was not possible to obtain a new transcriptomic library within the limits of the project. Future studies are planned to explore conditions under which OTA and CIT gene expression would be more clearly observed.

Our data illustrate the limits of relying on gene-metabolite associations based on similar accumulation patterns in metabolite production and gene expression. Relying on sequence similarity alone has also been used to identify a putative OTA-PKS in *Penicillium thymicola* DAOMC 180753, but did not provide definitive proof of correct gene annotation.^36^ A more rigorous approach to obtain definitive proof is to perform the commonly used gene disruption experiments or the more comprehensive full characterization of PKS enzymes as accomplished for the highly reducing polyketide synthase of Lovastatin.^37^ Both methods are the gold standard of determining enzyme and, thus, gene functions. Neither of these techniques was within the scope of our study. Instead, the unveiled OTA and CIT biosynthetic gene sequences were subjected to additional bioinformatic analysis. The complete sequences of the OTA-PKS from several OTA-producing fungal species were analyzed by Conserved Domain (Figure 5 and Supporting Information Figure S9).^13,14^ All sequences, including the one identified for *P. verrucosum* DAOMC 242724 (g09503), showed the same domain arrangement expected of a highly reducing polyketide synthase. All of the OTA-PKS enzymes used in the analysis appeared to contain a functioning ER and KR domain, thus classifying them as highly reducing polyketide synthases. As the OTA-PKS lacked a terminal TE domain, typically required to release the intermediate product, the OTA biosynthetic gene cluster contained a SnoaL cyclase encoding gene (*otaY*).^31^ A counterpart of *otaY* was identified in our study (g07681). Together with all of the other tailoring genes identified (Table 1), it is likely that the biosynthetic genes for OTA identified in our study are correct (please see Additional File 1). It is interesting to note that a putative OTA-PKS from a different strain of *P. verrucosum* was previously identified through gene disruption.^10^ The report did not provide a gene sequence, but did claim that the OTA-PKS of *P. verrucosum* and *P. nordicum* had different domain arrangements and that the OTA-PKS of *P. nordicum* did not contain a methyl transferase domain. Furthermore, the report asserted that the OTA-PKS of *P. verrucosum* had more in common with the CIT-PKS of *Monascus anka*. These claims contradict our findings, which determined that the OTA-PKS of *P. verrucosum* and *P. nordicum* are similar and that the OTA-PKS and CIT-PKS are distinct enzymes. The CIT-PKS has been extensively studied and was already classified as a nonreducing polyketide synthase, in contrast to the highly reducing polyketide synthase designation of the OTA-PKS.^12,15,32^ The corresponding sequences for the CIT-PKS from *P. verrucosum* DAOMC 242724 (g03741–42) were found to contain the PT domain, typically found in nonreducing polyketide synthases and the required SAM-dependent C-methyl transferase domain (Supporting Information Figure S11).

The underlying basis to explain why metabolite gene expressions were mitigated in shake culture is not known. It is generally known that as nutrients are depleted in the culture medium, secondary metabolite production is induced. Nutrient depletion-induced metabolite production has been documented in the case of *Fusarium graminearum*, whereby the onset of production of 3-or 15-acetyl-deoxynivalenol was marked by a concurrent reduction of glucose or amines in the growth medium.^38−40^ We surmised that this phenomenon was possibly at play in our study as well, whereby sensing a decrease in certain essential nutrients as the culture ages, *P. verrucosum* activated genes involved in metabolite production. In this study, double the volume of liquid medium was used in shake culture (100 mL), compared to 50 mL in stationary mode. It is possible that *P. verrucosum* was subjected to greater nutritional stress when grown in smaller volumes of liquid media. To ensure that the low concentration of OTA was not a one-off, the shake culture conditions were repeated. A lower OTA production was still observed, capping at 521 nM on Day 6, similar to a maximum OTA concentration of 587 nM reported in Figure 2b (Supporting Information Figure S18). An additional time point for OTA quantitation was collected on Day 7 for Figure 2b. Surprisingly, the three biological replicate liquid cultures harvested on Day 7 showed a dramatic drop in OTA concentration to 182 nM (Supporting Information Figure S19). Although not entirely definitive, it appeared that *P. verrucosum* DAOMC 242724, grown under the two-stage shake culture described here, experienced peak OTA production between 500 and 600 nM after 6 days of growth.

Temperature may also have played an important role in metabolite production. Since *P. verrucosum* DAOMC 242724 thrives in a temperate environment, it is possible that it prefers cooler growing conditions. The two-stage shake cultures were performed at a temperature of 25 °C, whereas the single-stage stationary cultures were followed at 20 °C. Overall, growing undisturbed, in a minimal volume of liquid medium, and at a cooler temperature appear to be optimal conditions for *P. verrucosum* DAOMC 242724 to produce more metabolites. It is interesting to note that *P. verrucosum* grown under shake and stationary culture have different physical attributes. In shake culture, *P. verrucosum* grew as individual spherical mycelial entities, which remained submerged in the liquid media throughout the growth period (Supporting Information Figure S20). In stationary mode, *P. verrucosum* grew as a single mycelial mat rapidly producing abundant aerial mycelia (Supporting Information Figure S21). In stationary PYMS media, less mycelial growth was observed but the majority of the mycelia was still aerial (data not shown). Altogether, our research illustrates how screening for more suitable culture conditions prior to obtaining transcriptomic data can be advantageous. Bioinformatic analyses of unveiled sequences can provide additional support to predict gene functions in the absence of gene disruption or functional characterization experiments.

## Supporting Information Available

Additional graphs, figures, bioinformatic results, and gene sequences (PDF)
