# Supplementary material for: Production of Ochratoxin A and Citrinin and the Expression of Their Biosynthetic Genes from Penicillium verrucosum in Liquid Culture
Source: ACS Omega. 2024 Apr 23;9(18):20368–77. doi: 10.1021/acsomega.4c00874 (PMC11080038; doi:10.1021/acsomega.4c00874)
Supplement: Supplementary file 1 — ao4c00874_si_001.pdf [file ao4c00874_si_001.pdf]

The Production of Ochratoxin A and Citrinin and the Expression of their Biosynthetic Genes from *Penicillium verrucosum* in Liquid Culture

Marc Sasseville,*[1] Hai D.T. Nguyen,[2] Simon Drouin[1] and Adilah Bahadoor*[3]

[1] Applied Genomics, Human Health Therapeutics, National Research Council, 6100 Royalmount Ave, Montreal, Quebec H4P 2R2, Canada

[2] Ottawa Research and Development Centre, Agriculture and Agri-Food Canada, 960 Carling Ave, Ottawa, Ontario, K1A 0C6, Canada

[3] Metrology, National Research Council, 1200 Montreal Road, Ottawa, Ontario, K1A 0R6, Canada

Corresponding authors: adilah.bahadoor@nrc-cnrc.gc.ca; marc.sasseville@nrc-cnrc.gc.ca

Table of Contents:

Figures	Page
**Figure S1.** The mapped genes of the transcriptome of *P. verrucosum* DAOMC 242724 as it produced OTA and CIT over 6 days.	S4
**Figure S2. (A)** The extracted ion chromatogram of a 13C6-OTA standard solution (m/z = 410.1096). **(B)** The extracted ion chromatogram of OTA (m/z = 404.0985) from a 10x-diluted sample.	S5
**Figure S3. (A**) Extracted ion chromatogram of a CIT standard solution (m/z = 251.09140). **(B)** Extracted ion chromatogram of CIT from a 20x-diluted LCMS sample (m/z = 251.09140).	S6
**Figure S4.** The LCMS values of 13C6-OTA, from the internal calibrant and CRM OTAL-1, obtained for each biological replicate (BR) during the quantitation of OTA in 2-stage shake culture.	S7
**Figure S5.** The LCMS values of 13C6-OTA, from the internal calibrant and CRM OTAL-1, obtained for each biological replicate (BR) during the quantitation of OTA in single stage YES medium + NH_4Cl in stationary phase.	S8
**Figure S6.** The LCMS values of 13C6-OTA, from the internal calibrant and CRM OTAL-1, obtained for each biological replicate (BR) during the quantitation of OTA in single stage PYMS medium in stationary phase.	S9
**Figure S7**. The production profile of CIT produced in stationary YES medium supplement with NH_4Cl, showing all the timepoints monitored by LC-MS during sampling over 42-days.	S10
**Figure S8.** The production profile of CIT produced in stationary PYMS, showing all the timepoints monitored by LC-MS during sampling over 42-days.	S11
**Figure S9**. The domains of the OTA PKS obtained from Conserved Domain shows that it is a highly reducing polyketide synthase.	S12
**Figure S10.** The domains of the CIT PKS of *M. purpureus* obtained from Conserved Domain.	S13
**Figure S11.** The domains of the CIT PKS (g037142-42) of *P. verrucosum* DAOMC 242724 obtained from Conserved Domain	S14
**Figure S12.** PCA analysis showed that the genes clustered in two groups, day 1-2 and day 3-6.	S15
**Figure S13.** Up-regulated and down-regulated gene clusters when compared to Day 1.	S16
**Figure S14.** Typical expression profile of genes from Cluster 1, a set of 67 genes, whose expression was repressed by a Log2 factor of -2 to -4 from Day 1.	S17
**Figure S15.** Typical expression profile of genes from Cluster 2, a set of 45 genes, whose expression was increased by a Log2 factor of 6 to 8 from Day 1.	S18
**Figure S16**. Typical expression profile of genes from Cluster 3, a set of 109 genes whose expression was increased by a Log2 factor of 2 to 4 from Day 1.	S19
**Figure S17.** Typical expression profile of genes from Cluster 4, a set of 43 genes whose expression was reduced by a Log2 factor of 6 to 8 from Day 1.	S20
**Figure S18.** The production of OTA in 2-stage shake culture in YES medium repeated a second time	S21
**Figure S19.** The production of OTA drops significantly on Day 7 in 2-stage shake culture in YES medium	S22

**Figure S20.** The appearance of *P. verrucosum* DAOMC 242724 over 5 days of growth in 100 mL of YES media in shake culture.	S23
**Figure S21.** The appearance of *P. verrucosum* DAOMC 242724 over 42 days of growth in 50 mL of YES media supplemented with NH_4Cl in stationary culture.	S24
**Tables**	
**Table S1.** Trinity software *de novo* transcriptome analysis of *P verrucosum* DAOMC 242724 RNA library as it produced OTA and CIT over six days.	S25
**Table S2.** Alignment of coding transcripts from 686 *Penicillium* species from European Nucleotide Archive (ENA) database against *P verrucosum* genome (LAKW02001000.1)	S26
**Table S3**. Trinity-predicted OTA biosynthetic genes BLASTn alignment scores with comparable genes from the OTA biosynthetic gene cluster of from *P nordicum* DAOMC 185683.	S27
**Table S4.** Trinity-predicted OTA biosynthetic genes BLASTn alignment scores with highly similar sequences from the whole genome of *P verrucosum* BFE808.	S28
**Table S5.** Trinity-predicted CIT biosynthetic genes BLASTn alignment scores with comparable genes from the CIT biosynthetic gene clusters of *M purpureus and M. aurantiacus*.	S29
**Table S6.** Trinity-predicted CIT biosynthetic genes BLASTn alignment scores with highly similar sequences from the whole genome of *P verrucosum* BFE808.	S30
**Table S7.** Proteins sharing > 80% similarity with OTA-PKS from *P. verrucosum* 242724	S31
**Table S8.** Proteins sharing > 80% similarity with CIT-PKS from *P. verrucosum* DAOMC 242724	S32
**Table S9.** OTA genes identified by Trinity Software from RNASeq library and on the genome of BFE808.	S33
**Table S10.** CIT genes identified by Trinity Software from RNASeq library and on the genome of BFE808.	S34
**Gene Sequences**	
OTA-PKS gene sequence (otaA - g09503)	S35-S36
NRPS (otaB – g07679)	S37-S38
Cyp450 (otaC – g07680)	S39
Halogenase (otaD – g07677)	S40
BZip Transcription factor (otaR1 – g07678)	S41
Snoal Cyclase (otaY – g07681)	S42
CIT-PKS (citsS – g03741-42)	S43-S44
Serine hydrolase (ctnB or citA – g06948)	S45
MFS transporter (ctnC – g03740)	S46
Shortchain dehydrogenase (ctnE – g06947)	S47
Transcriptional factor (ctnR – g06944)	S48
Oxidoreductase (ctnD or citC – g06943)	S49

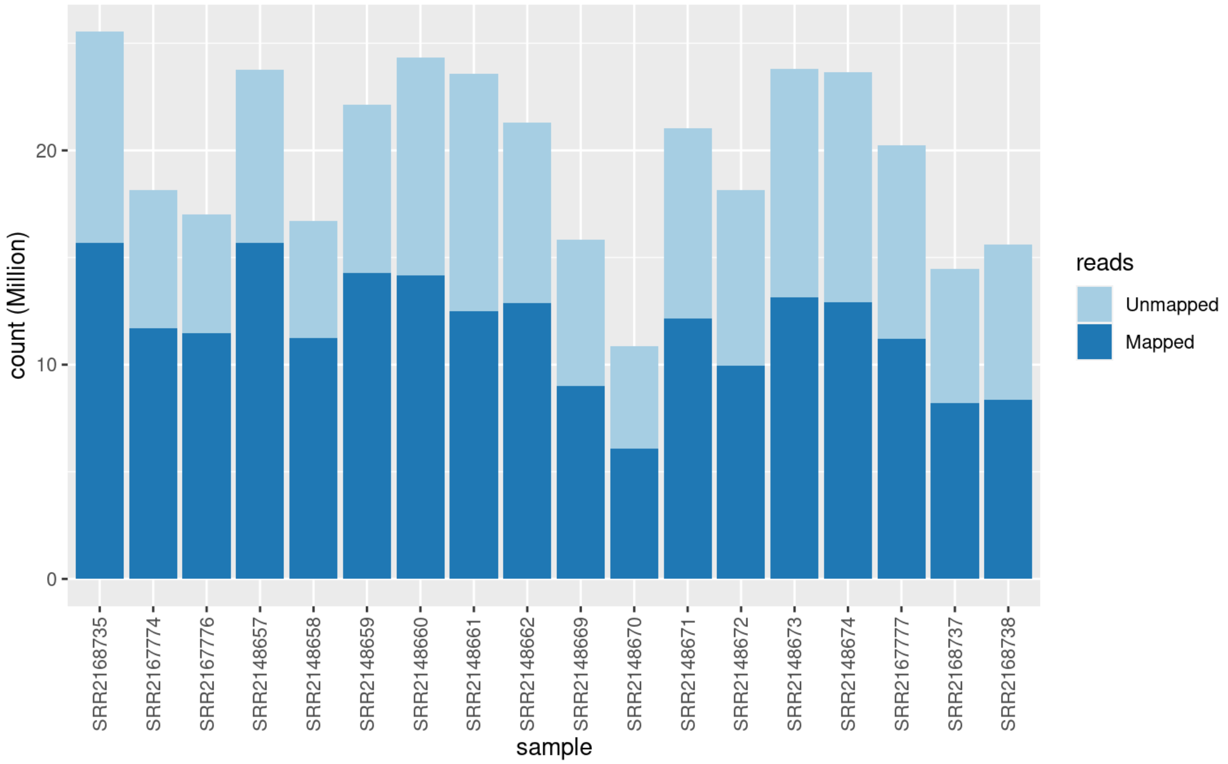

Figure S1. The mapped genes of the transcriptome of *P verrucosum* DAOMC 242724 as it produced OTA and CIT over 6 days.

A)

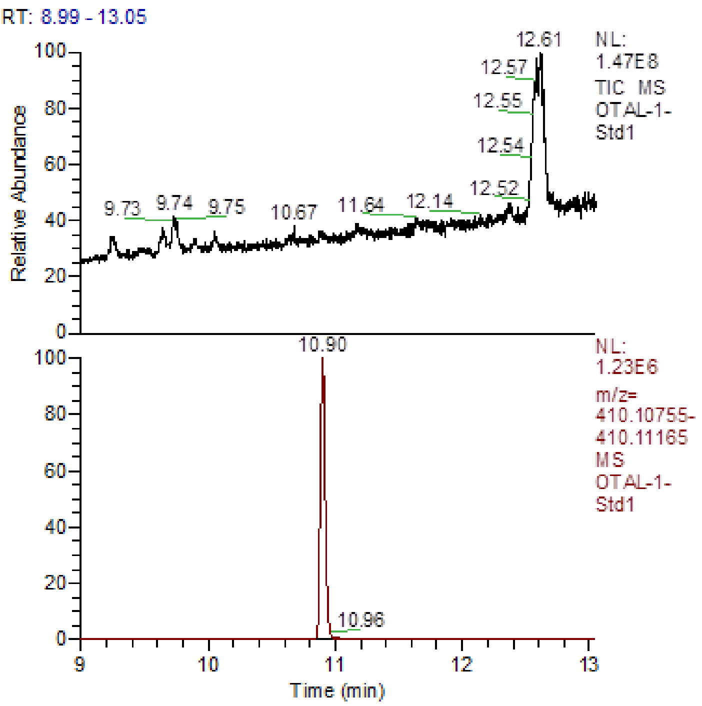

B)

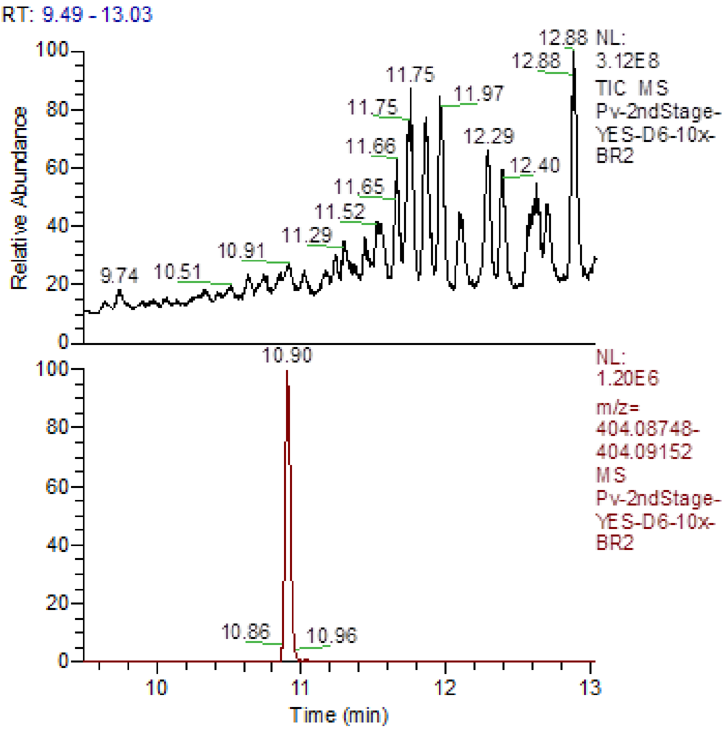

Figure S2. (A) The extracted ion chromatogram of a 13C6-OTA standard solution (m/z = 410.1096). **(B)** The extracted ion chromatogram of OTA (m/z = 404.0985) from a 10x-diluted sample.

A)

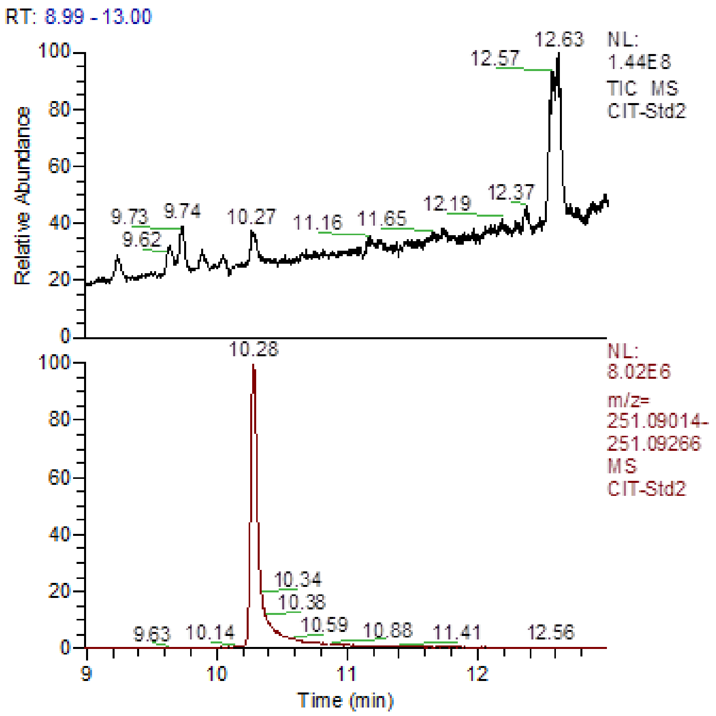

B)

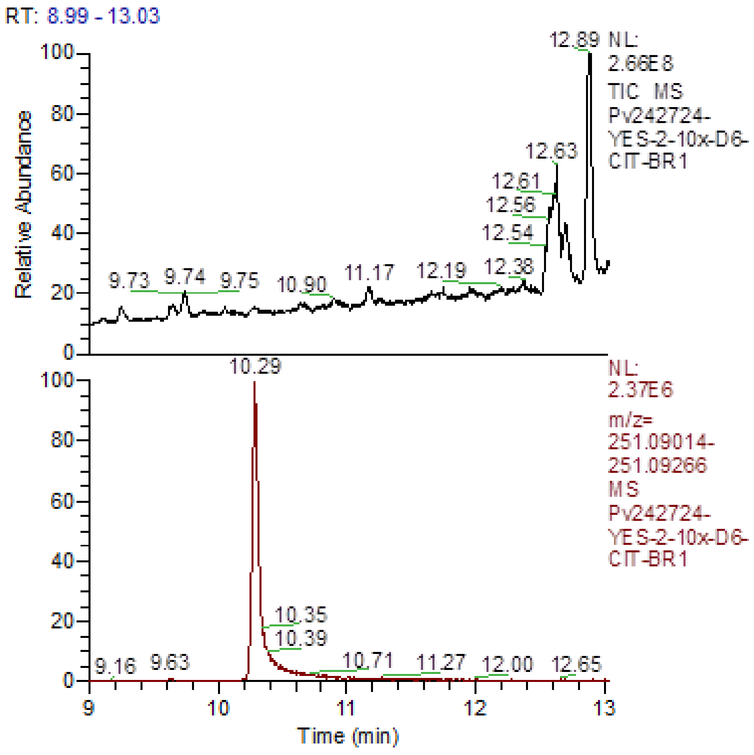

Figure S3. (A) Extracted ion chromatogram of a CIT standard solution (m/z = 251.09140). **(B)** Extracted ion chromatogram of CIT from a 20x-diluted LCMS sample (m/z = 251.09140).

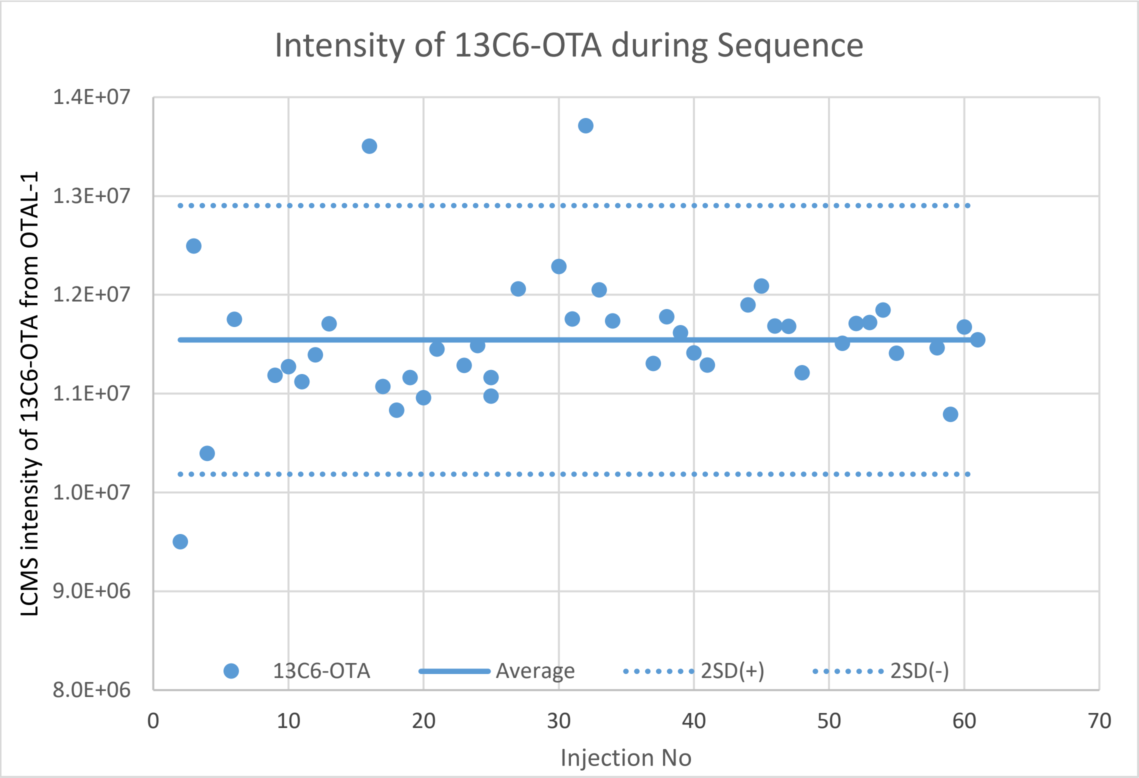

Figure S4. The LCMS values of 13C6-OTA, from the internal calibrant and CRM OTAL-1, obtained for each biological replicate (BR) during the quantitation of OTA in 2-stage shake culture are shown. Most individual readings are within the two standard deviation of the mean, thus showing that the volume of OTAL-1 added to each sample was more or less similar as no significant deviation from the mean was observed. For those that lay outside the two standard deviation of the mean, the values were not significantly far off the mean to significantly impact the average values calculated from the four individual biological replicates obtained for each time-point.

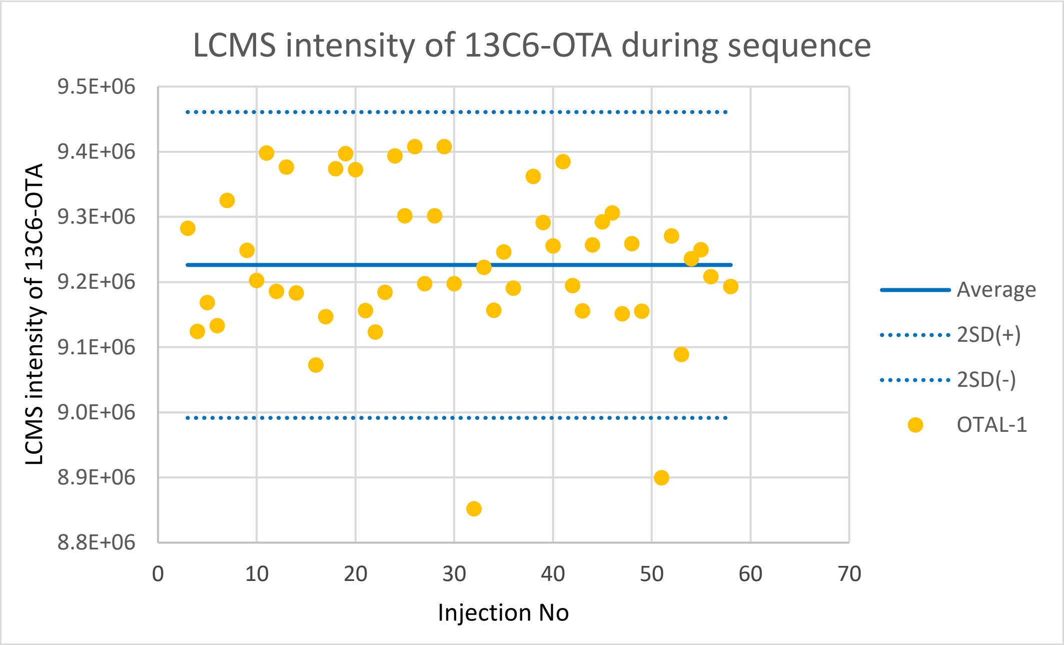

Figure S5. The LCMS values of 13C6-OTA, from the internal calibrant and CRM OTAL-1, obtained for each biological replicate (BR) during the quantitation of OTA in single stage YES medium + NH_4Cl in stationary phase are shown. Most individual readings are within the two standard deviation of the mean, thus showing that the volume of OTAL-1 added to each sample was more or less similar as no significant deviation from the mean was observed. For those that lay outside the two standard deviation of the mean, the values were not significantly far off the mean to significantly impact the average values calculated from the four individual biological replicates obtained for each time-point.

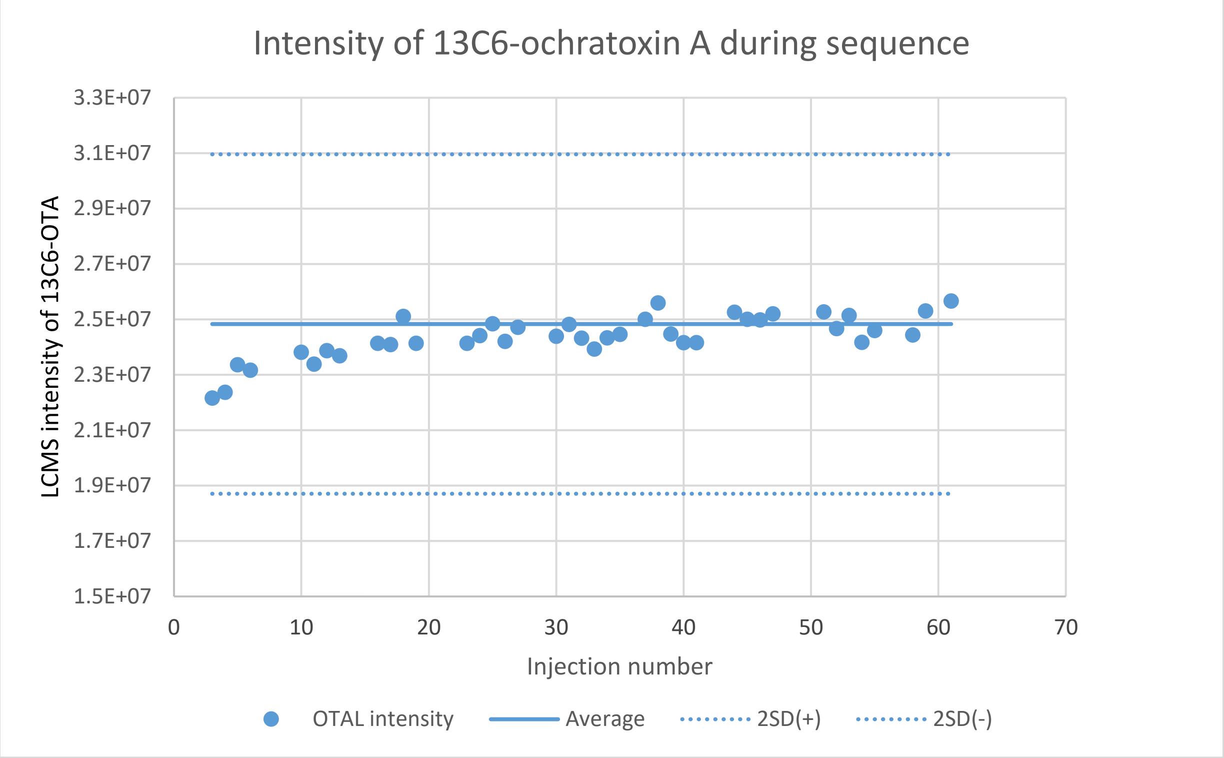

Figure S6. The LCMS values of 13C6-OTA, from the internal calibrant and CRM OTAL-1, obtained for each biological replicate (BR) during the quantitation of OTA in single stage PYMS medium in stationary phase are shown. All individual readings are within the two standard deviation of the mean, thus showing that the volume of OTAL-1 added to each sample was more or less similar as no significant deviation from the mean was observed.

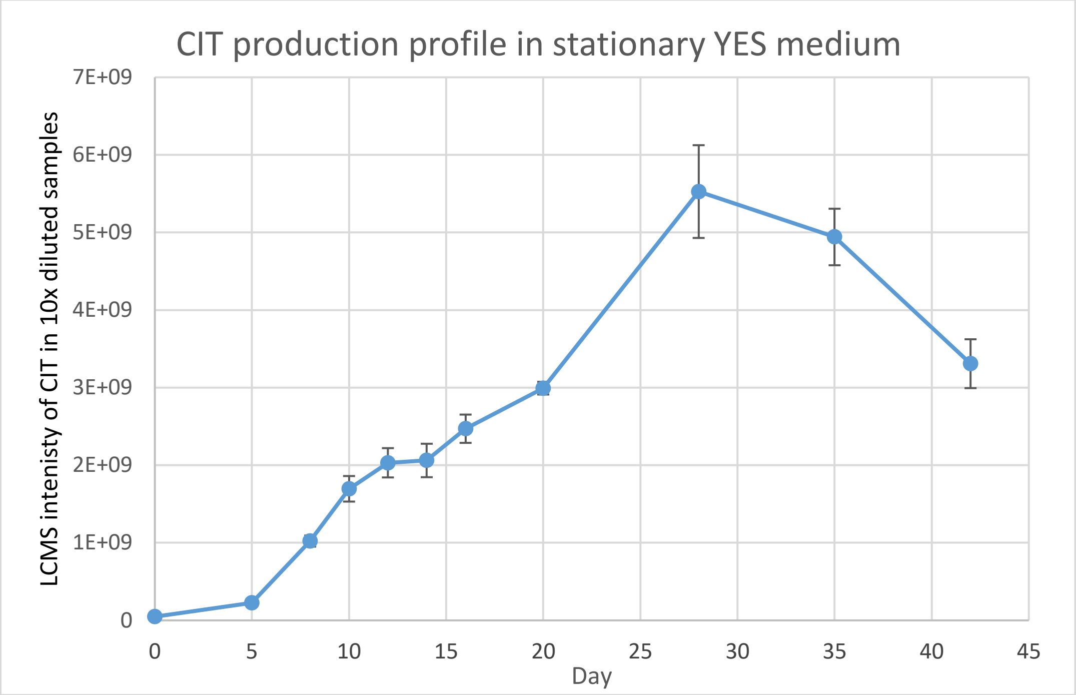

Figure S7. The production profile of CIT produced in stationary YES medium supplement with NH_4Cl, showing all the timepoints monitored by LC-MS during sampling over 42-days.

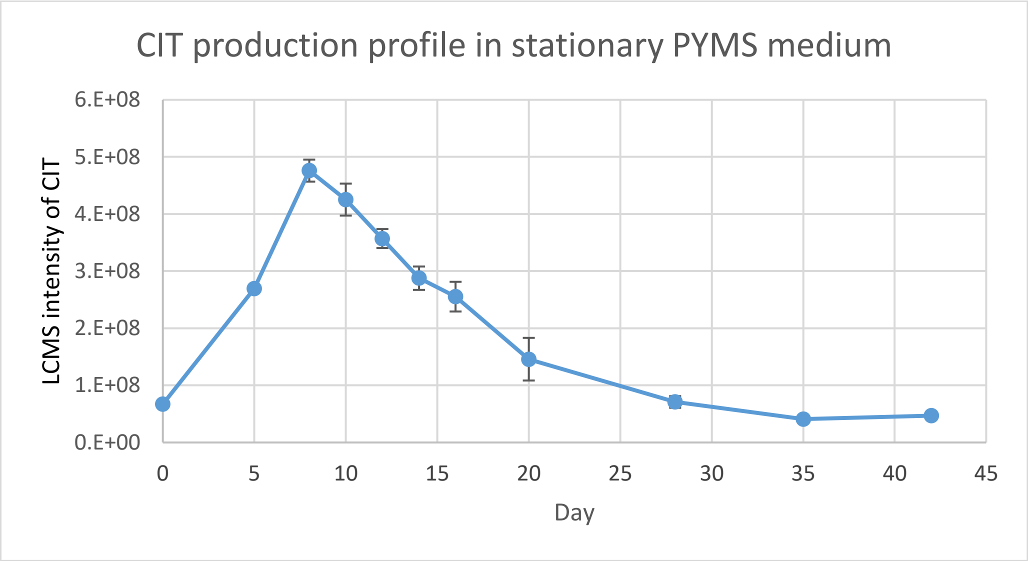

Figure S8. The production profile of CIT produced in stationary PYMS, showing all the timepoints monitored by LC-MS during sampling over 42-days.

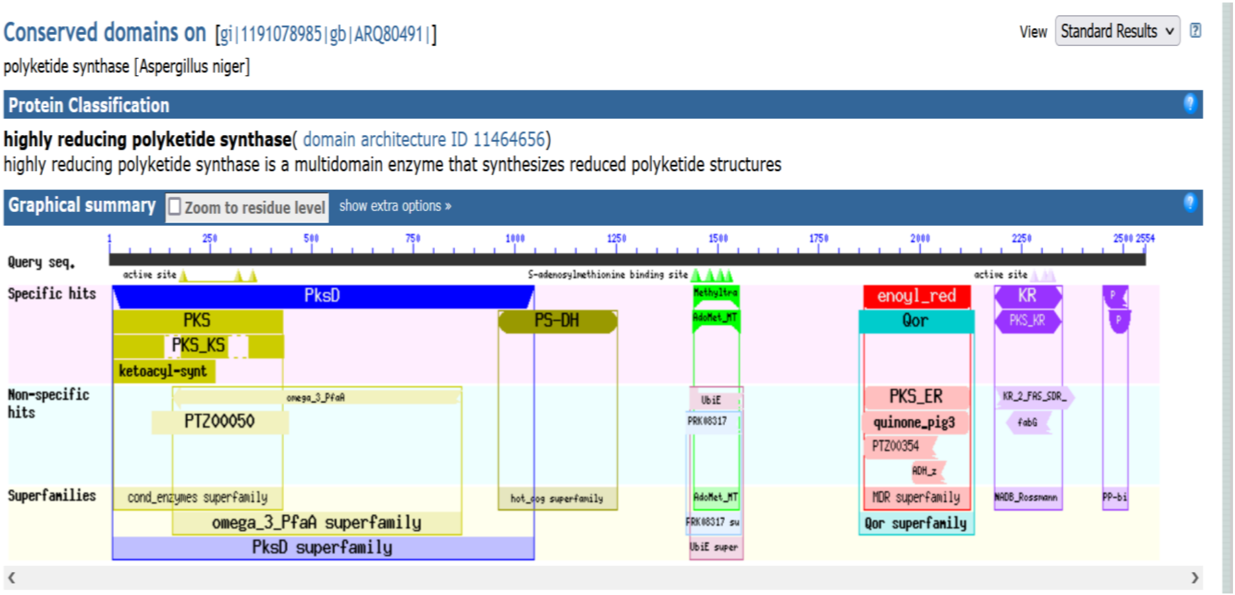

Figure S9. The domains of the OTA PKS of *A. niger* (KX519720.1) obtained from Conserved Domain shows that it is a highly reducing polyketide synthase. The exact same domain arrangement was obtained for the OTA-PKS of *A. steynii* (KJ395384.1), *A. westerdijkiae* (MW526250.1), *A. carbonarius* (MG701890.1), *P. nordicum* (MG701895.1) and *P. verrucosum* (g09503).

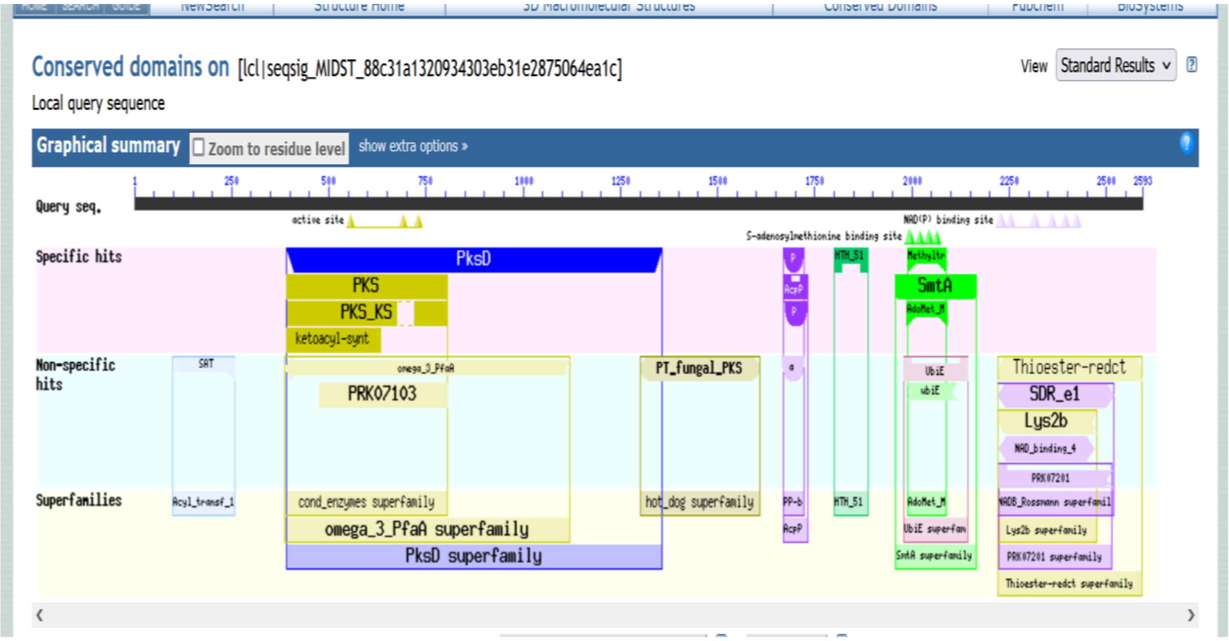

Figure S10. The domains of the CIT PKS of *M. purpureus* obtained from Conserved Domain. The sequence used to determine the domain identities was obtained from Storm et al., **2017**, *Cell Chemical Biology* 24, 316–325, which provided an updated amino sequence of the CIT PKS *M. purpureus* (AB167465.1).

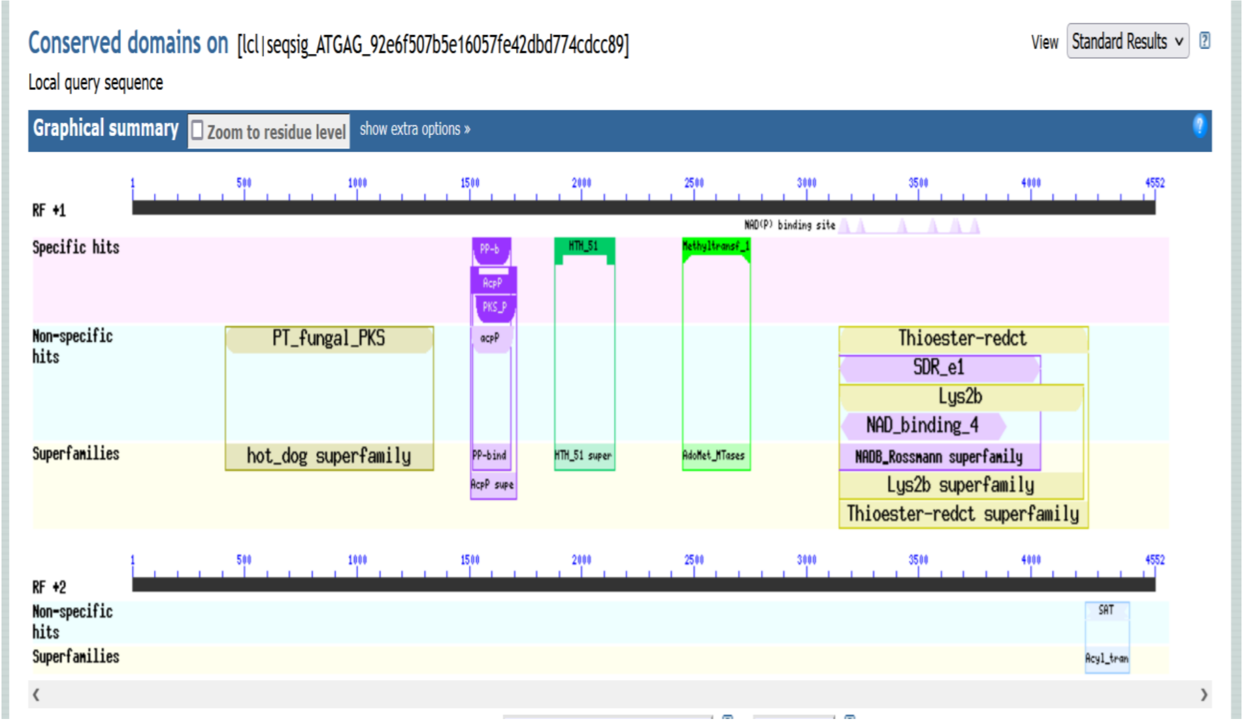

Figure S11. The domains of the CIT PKS (g037142-42) of *P. verrucosum* DAOMC 242724 obtained from Conserved Domain shows the product template domain (PT), the phosphopantetheine arm (PP), the C-methyl transferase domain (CMT) and the thioester domain (TE). The sequence is incomplete lacking the starter acyl transferase unit (SAT)and the ketoacyl synthase (KS) domain.

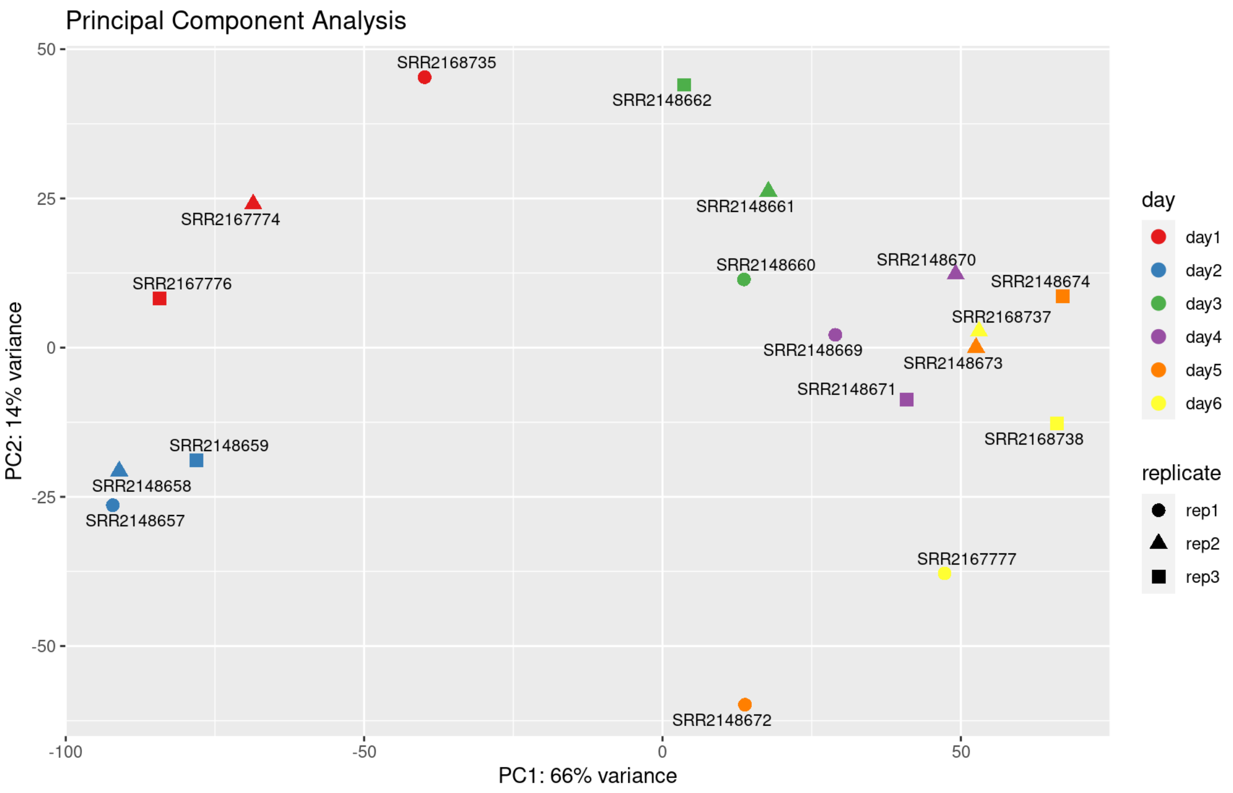

Figure S12. PCA analysis showed that the genes clustered in two groups, day 1-2 and day 3-6.

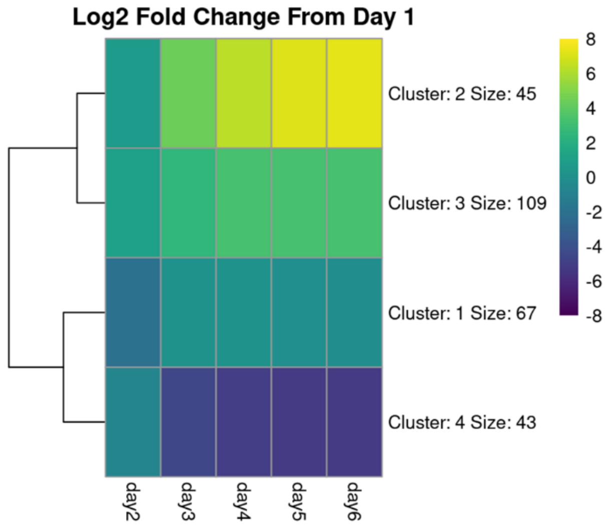

Figure S13. Up-regulated and down-regulated gene clusters when compared to Day 1.

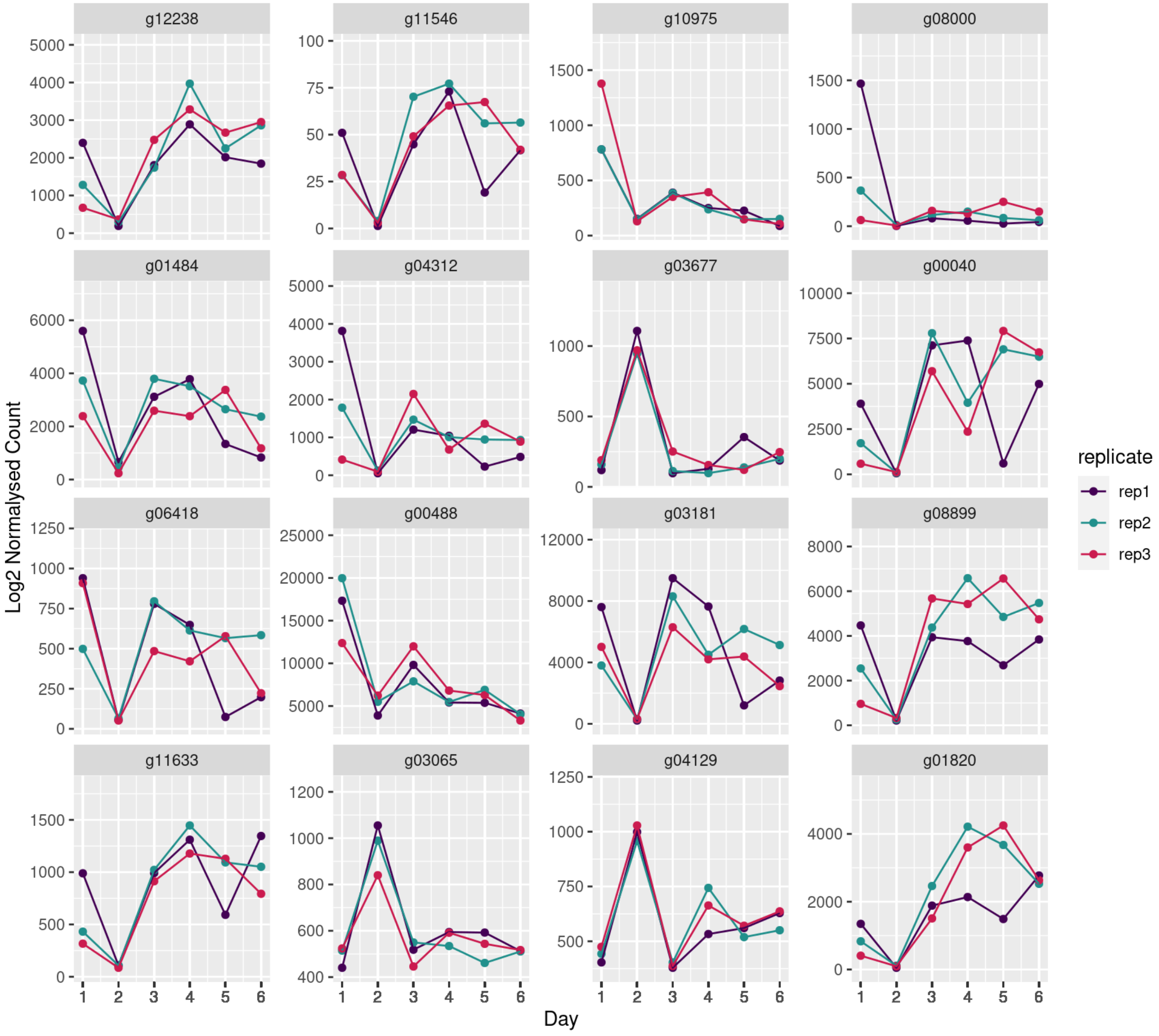

Figure S14. Typical expression profile of genes from Cluster 1, a set of 67 genes, whose expression was repressed by a Log2 factor of -2 to -4 from Day 1.

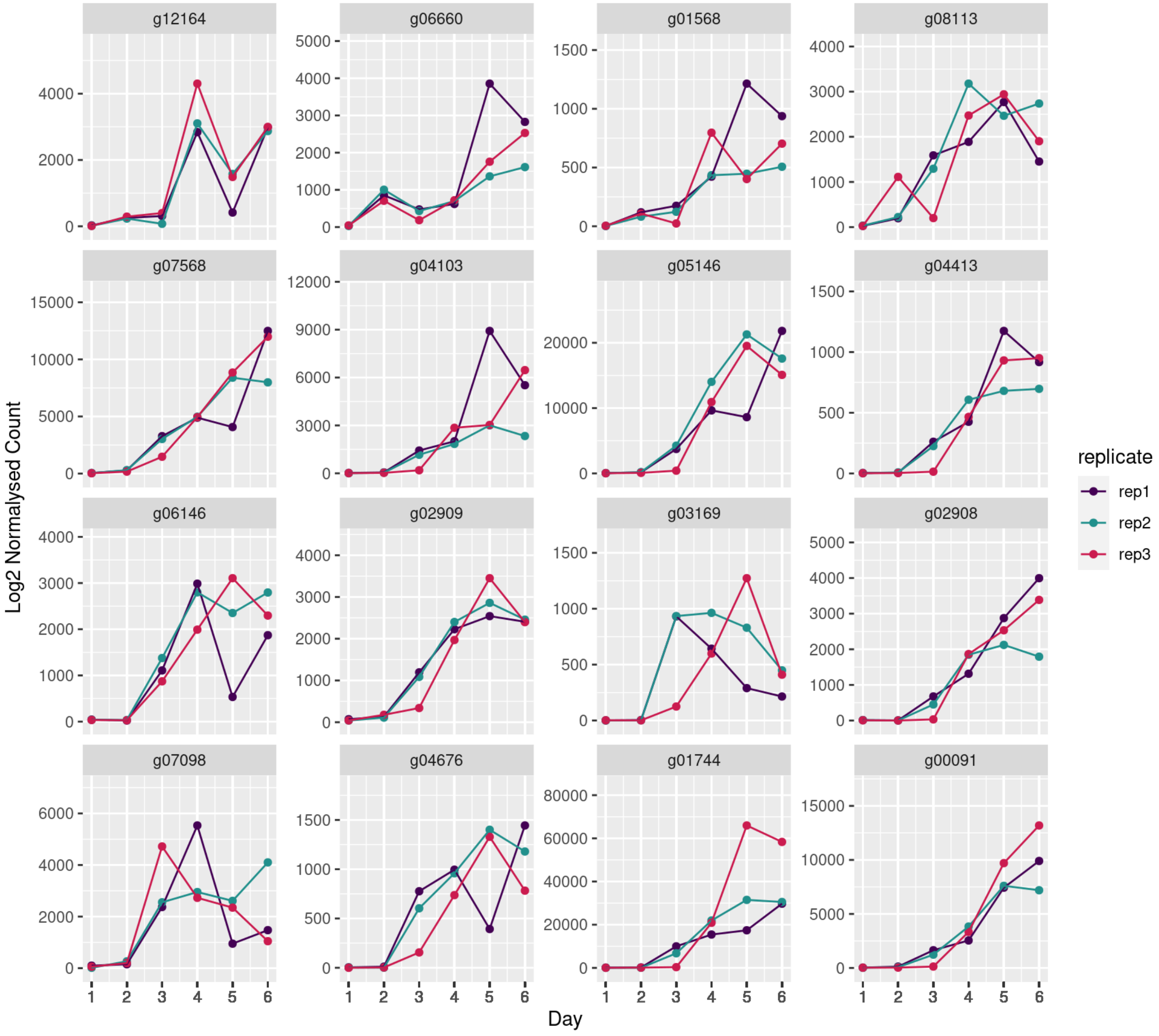

Figure S15. Typical expression profile of genes from Cluster 2, a set of 45 genes, whose expression was increased by a Log2 factor of 6 to 8 from Day 1.

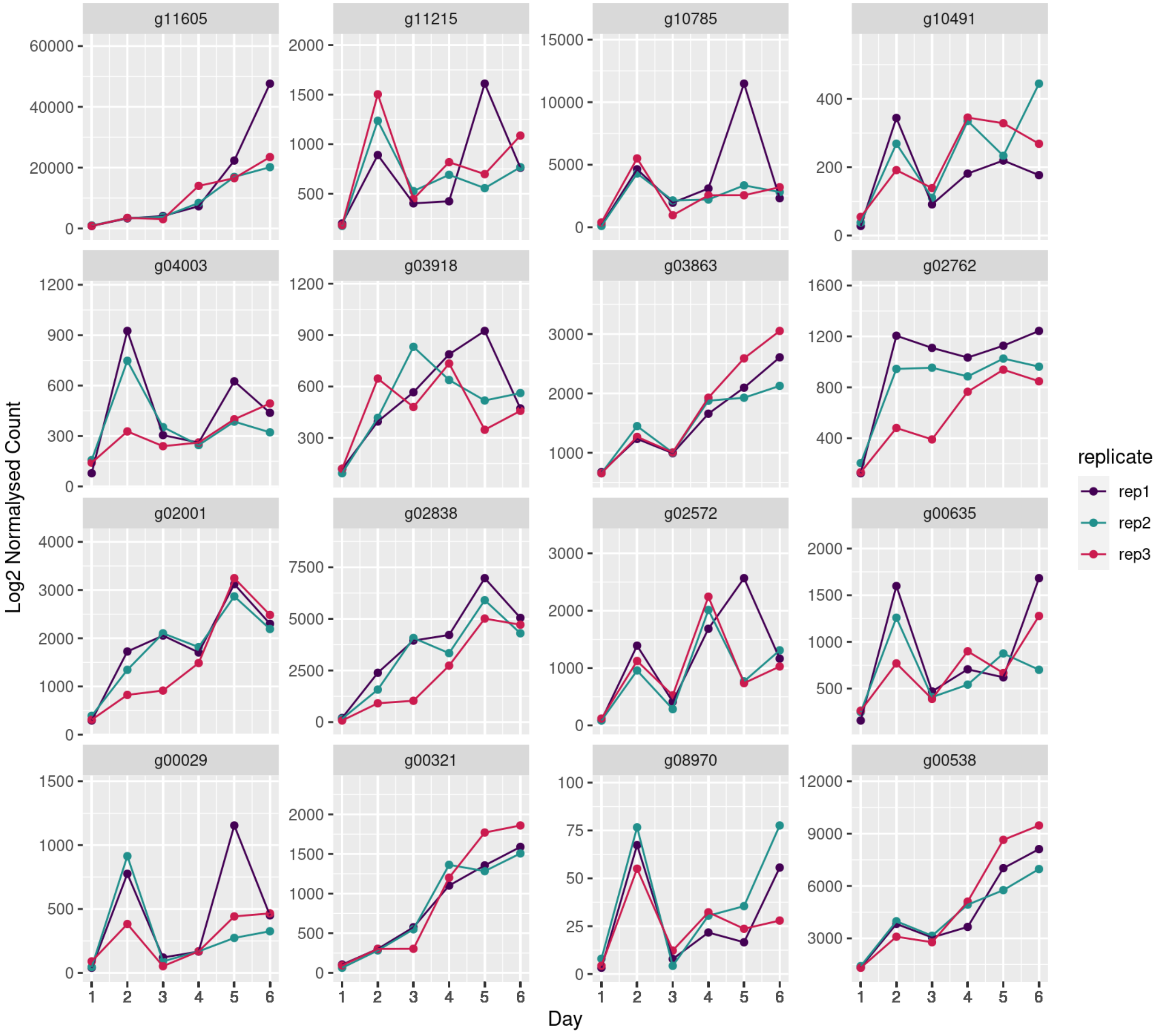

Figure S16. Typical expression profile of genes from Cluster 3, a set of 109 genes whose expression was increased by a Log2 factor of 2 to 4 from Day 1.

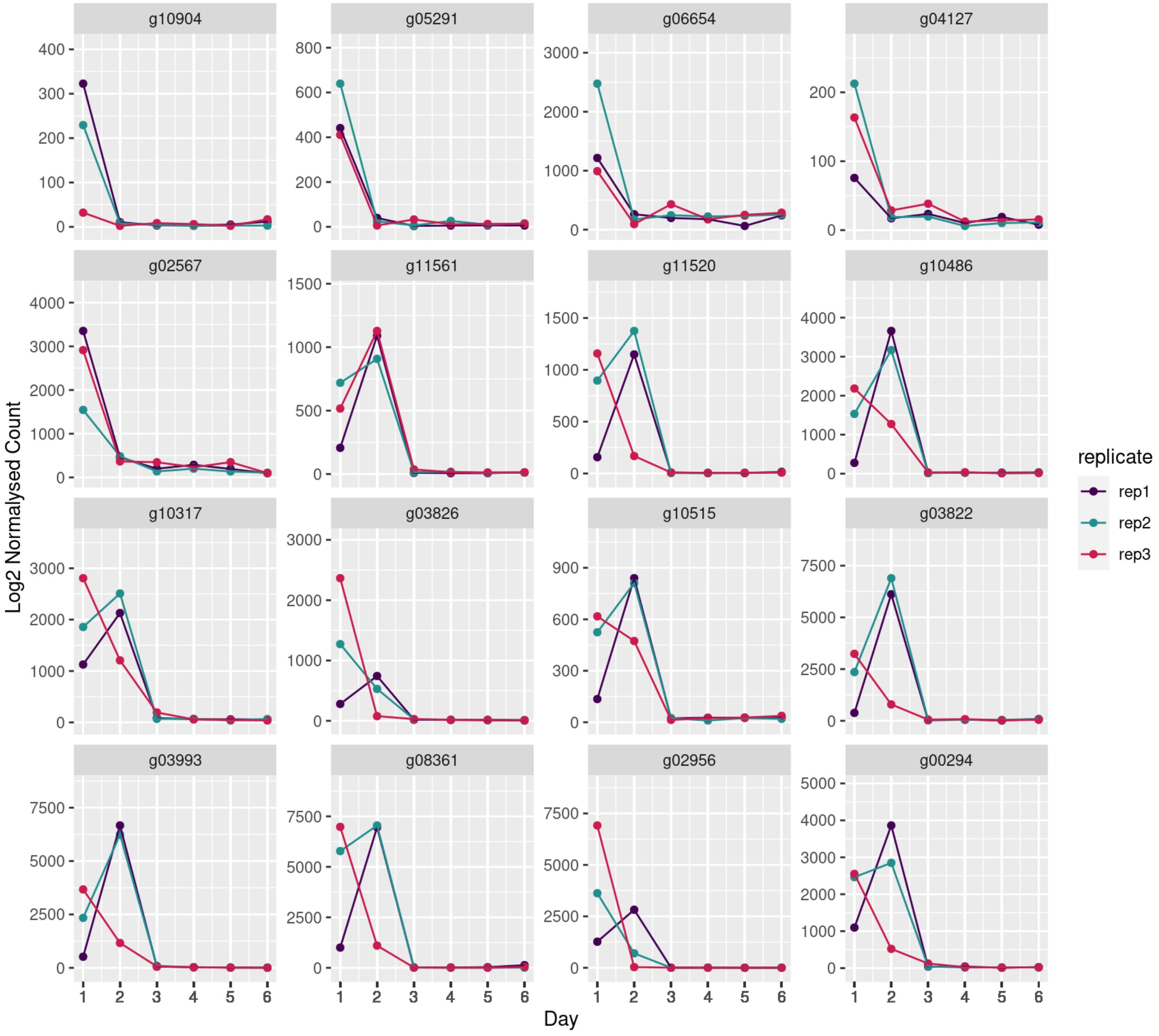

Figure S17. Typical expression profile of genes from Cluster 4, a set of 43 genes whose expression was reduced by a Log2 factor of 6 to 8 from Day 1.

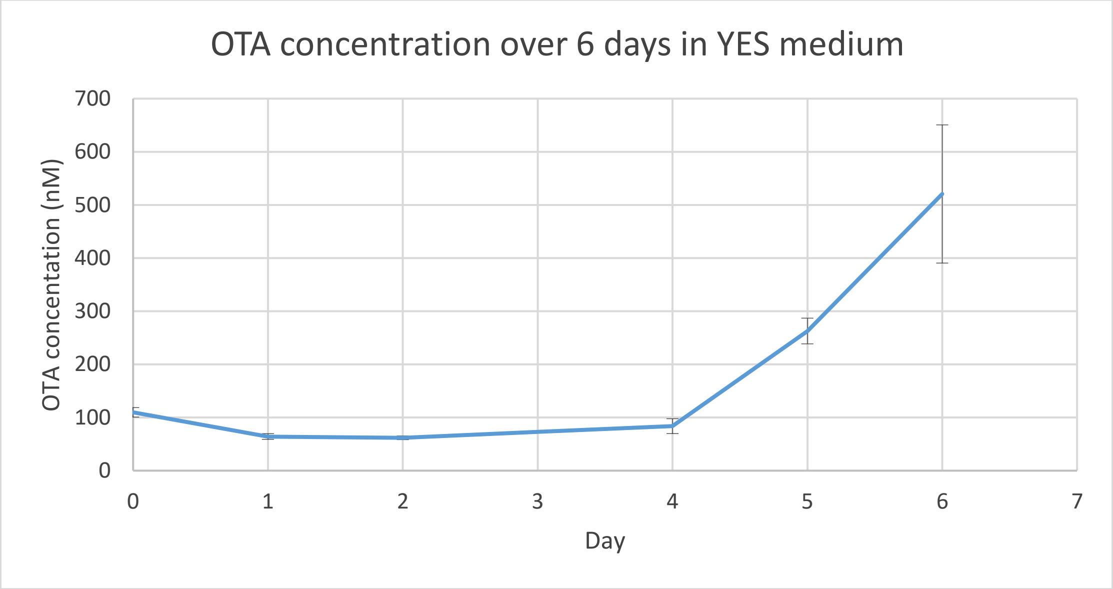

Figure S18. The production of OTA in 2-stage shake culture in YES medium repeated a second time

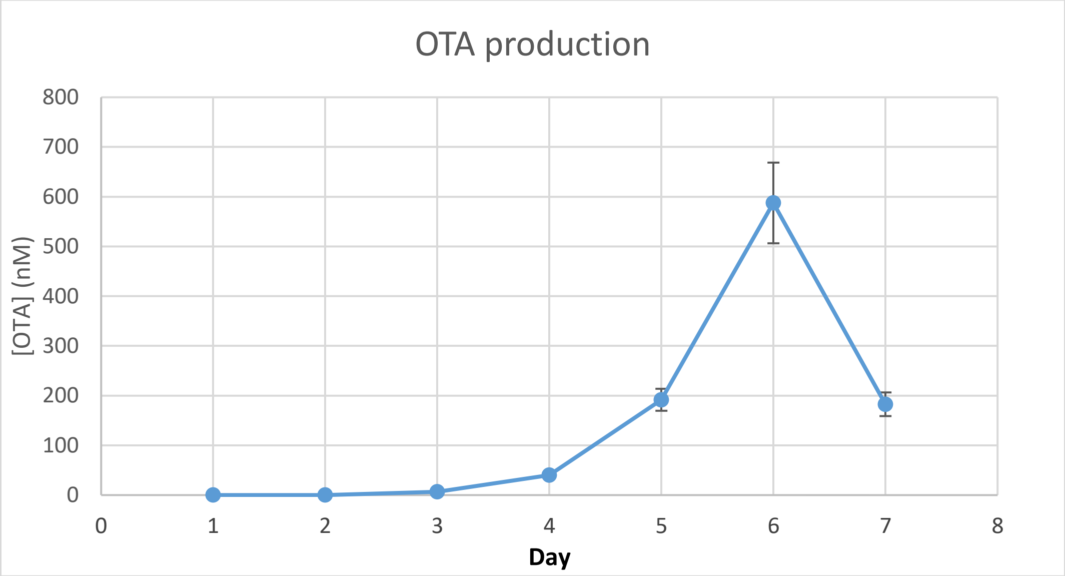

Figure S19. The production of OTA drops significantly on Day 7 in 2-stage shake culture in YES medium

Day 1: 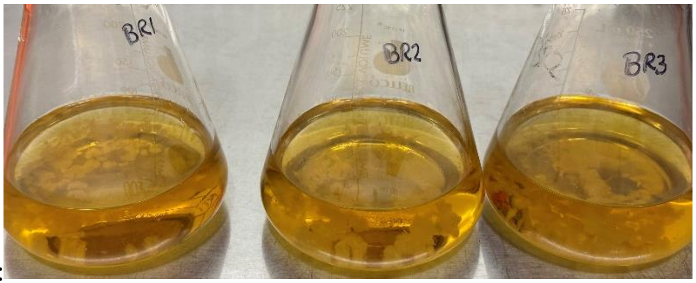

Day 2: 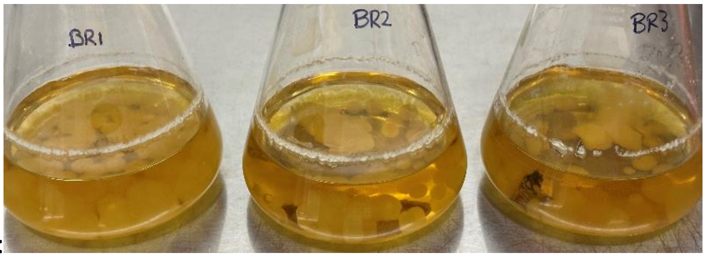

Day 3: 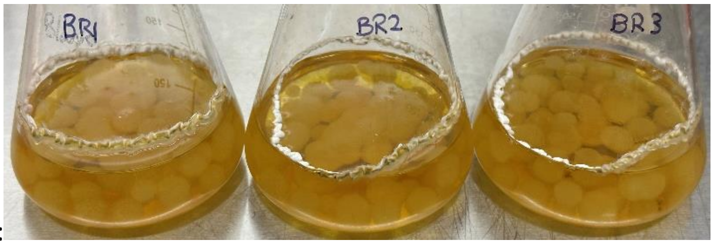

Day 4: 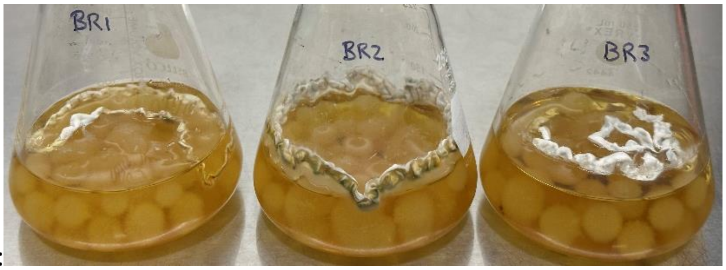

Day 5: 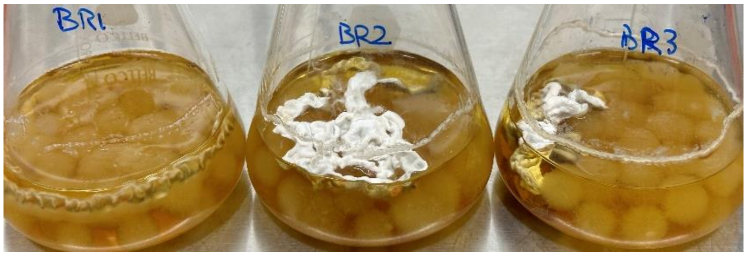

Figure S20. The appearance of *P. verrucosum* DAOMC 242724 over 5 days of growth in 100 mL of YES media in shake culture.

Day 5: 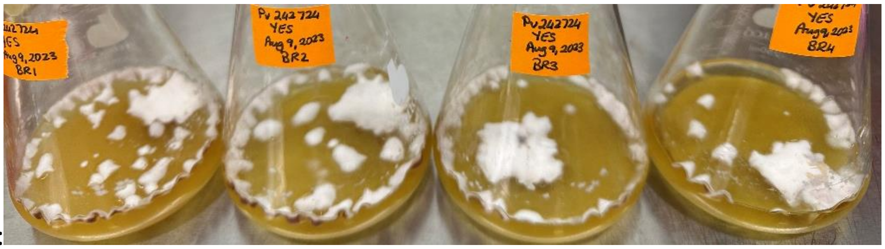

Day 8: 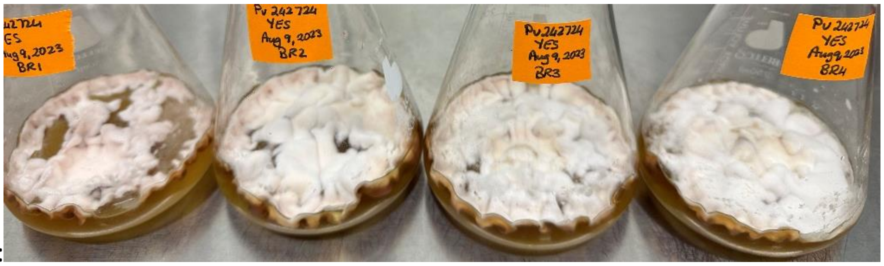

Day 10: 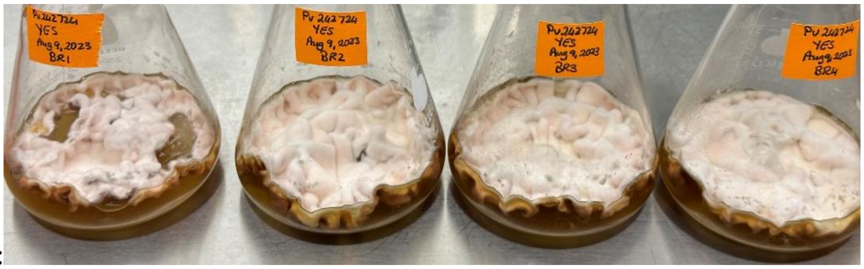

Day 16: 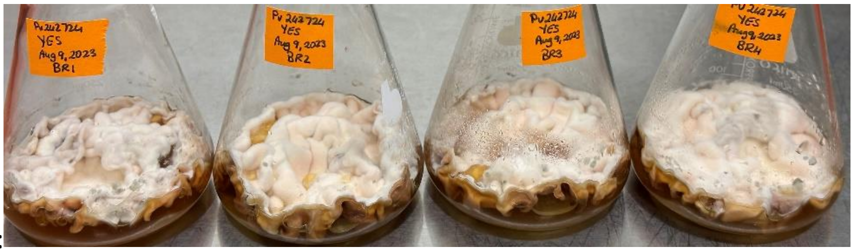

Day 28: 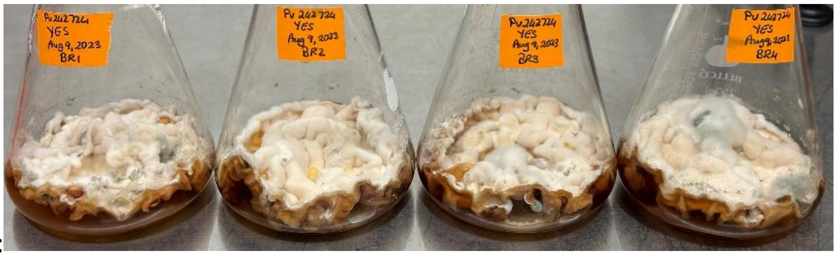

Day 42: 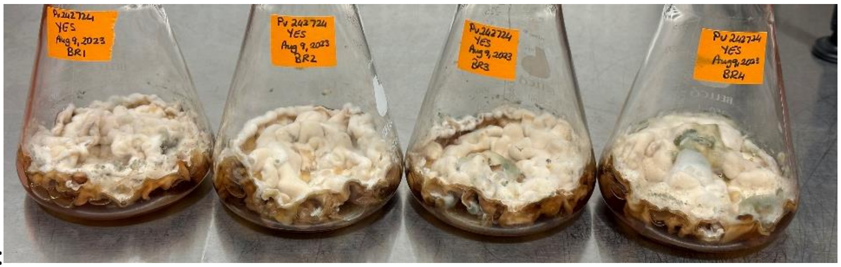

Figure S21. The appearance of *P. verrucosum* DAOMC 242724 over 42 days of growth in 50 mL of YES media supplemented with NH_4Cl in stationary culture.

Table S1. Trinity software *de novo* transcriptome analysis of *P. verrucosum* DAOMC 242724 RNA library as it produced OTA and CIT over six days.

Accession number of transcriptomic reads	Day	Biological Replicates	Total Reads
SRR2168735	day1	rep1	25 538 222
SRR2167774	day1	rep2	18 136 397
SRR2167776	day1	rep3	17 022 025
SRR2148657	day2	rep1	23 747 050
SRR2148658	day2	rep2	16 712 740
SRR2148659	day2	rep3	22 151 528
SRR2148660	day3	rep1	24 348 307
SRR2148661	day3	rep2	23 573 515
SRR2148662	day3	rep3	21 303 878
SRR2148669	day4	rep1	15 817 717
SRR2148670	day4	rep2	10 856 960
SRR2148671	day4	rep3	21 027 463
SRR2148672	day5	rep1	18 147 794
SRR2148673	day5	rep2	23 789 198
SRR2148674	day5	rep3	23 649 975
SRR2167777	day6	rep1	20 246 851
SRR2168737	day6	rep2	14 481 581
SRR2168738	day6	rep3	15 590 886

Table S2. Alignment of coding transcripts from 686 *Penicillium* species from European Nucleotide Archive (ENA) database against *P. verrucosum* BFE808genome (LAKW00000000.2)

Result	Count	Bases	Taxon descendants count	Bases
**Analysis**	0	0 bp	6	0 bp
**Assembly**	0	0 bp	158	5 GB
**Coding**	0	0 bp	229 663	295 MB
**Experiment**	21	76 GB	1202	4 TB
**Genome assembly coding set**	0	0 bp	97	3 GB
**Non-coding**	0	0 bp	16 002	4 MB
**Run**	21	76 GB	1363	4 TB
**Sample**	21	0 bp	1119	0 bp
**Sequence**	3	3 kB	52 367	701 MB
**Study (Analysis)**	0	0 bp	2	0 bp
**Study**	9	76 GB	297	3 TB
**Project**	5	0 bp	367	0 bp
**Transcriptome assembly contig set**	0	0 bp	5	276 MB

Table S3. Trinity-predicted OTA biosynthetic genes BLASTn alignment scores with comparable genes from the OTA biosynthetic gene cluster of *P. nordicum* DAOMC 185683.

Trinity genes	CDS and function of OTA gene from *P nordicum* DAOMC 185683	Max Score	Total Score	Query Cover	E value	Per. ident	Acc. Len	Accession number
g09503	CDS: (join(14698..14989,15052..15320,15386..16664,16725..17851, 17897..19513,19567..19628,19676..21488,21542..21862, 21923..22759)<br>/codon_start=1<br>/product="**polyketide synthase**"<br>/protein_id="AUS29495.1"	3088	12185	99%	0	97.26	8062	MG7011895.1
g07680	CDS: join(5446..5674,5742..6613,6665..6690,6745..6829,6877..7040,7108..7276)<br>/codon_start=1<br>/product="**cytochrome P450 monooxygenase**"<br>/protein_id="AUS29493.1	1519	2692	98%	0	98.05	1831	MG7011895.1
g07679	CDS: join(8083..8436,8486..13774)<br>/codon_start=1<br>/product="**non-ribosomal peptide synthetase**"<br>/protein_id="AUS29494.1"	9114	9689	100%	0	97.77	5692	MG7011895.1
g07878	CDS: join(3841..4429,4480..4607))<br>/codon_start=1<br>/product="**bZIP transcription factor**" /protein_id="AUS29492.1"	989	1209	100%	0	96.94	767	MG7011895.1
g07677	CDS: join(1..269,344..883,952..1359,1404..1443,1499..1595,1648..1772))<br>/codon_start=1<br>/product="**halogenase**"<br>/protein_id="AUS29491.1"	946	2106	82%	0	97.98	1359	MG7011895.1
g07681	<1..>353<br>/gene="otaY<br>/note="**cyclization of ochratoxin A polyketide backbone**"<br>/codon_start=1<br>/product="polyketide cyclase"	630	630	91%	0	98.87	353	MT706049.1

Table S4. Trinity-predicted OTA biosynthetic genes BLASTn alignment scores with highly similar sequences from the whole genome of *P. verrucosum* BFE808.

Trinity genes	Max Score	Total Score	Query Cover	E value	Per. ident	Acc. Len	Location on *P. verrucosum* BFE808 genome (LAKW02001000.1)
g09503	3354	13037	99%	0	99.89	51498	Penicillium verrucosum strain BFE808 Contig1009, whole genome shotgun sequence
g07680	1613	2821	98%	0	100	51498	Penicillium verrucosum strain BFE808 Contig1009, whole genome shotgun sequence
g07679	9762	10413	100%	0	99.98	51498	Penicillium verrucosum strain BFE808 Contig1009, whole genome shotgun sequence
g07878	1199	1199	100%	0	93.48	51498	Penicillium verrucosum strain BFE808 Contig1009, whole genome shotgun sequence
g07677	1002	2754	100%	0	99.82	51498	Penicillium verrucosum strain BFE808 Contig1009, whole genome shotgun sequence
g07681	715	715	100%	0	100	51498	Penicillium verrucosum strain BFE808 Contig1009, whole genome shotgun sequence

Table S5. Trinity-predicted CIT biosynthetic genes BLASTn alignment scores with comparable genes from the CIT biosynthetic gene clusters of *M purpureus and M. aurantiacus*. ([a] *M. purpureus*; [b] *M. aurantiacus*)

Trinity genes	CDS and function of OTA gene from *Monascus*	Max Score	Total Score	Query Cover	E value	Per. ident	Acc. Len	Accession number
g06943	complement(join(396..2027,2076..2312)) /gene="ctnD" /note="**oxidoreductase**" /codon_start=1 /product="CtnD" /protein_id="ACA34720.1"	1205	1641	92%	0	82.04[b]	1917	EU309474.1
g06944	join(2594..2831,2891..3164,3217..3724,3801..4289, 4378..4599) /gene="ctnR" /codon_start=1 /product="**citrinin biosynthesis transcriptional activator CtnR**” /protein_id="BAE95337.1"	1546	1546	86	0	85.18[a]	1731	AB243687.1
g06947	complement(join(2522..3327,3443..3464)) /gene="ctnE" /note="**dehydrogenase**" /codon_start=1 /product="CtnE" /protein_id="ACA34721.1"	976	976	97%	0	87.19[b]	943	EU309474.1
g06948	6113..7054 /gene="ctnB" /codon_start=1 /product="**citrinin biosynthesis oxydoreductase CtnB**" /protein_id="BAE95339.1"	787	787	98%	0	85.01[a]	942	AB243687.1
g03740	complement(join(16867..17033,17084..18167,18230..18544)) /gene="ctnC" /codon_start=1 /product="**citrinin biosynthesis transporter CtnC**" /protein_id="BAE95340.1"	859	2888	99	0	87.43[a]	3104	AB243687.1
g03741-g03742	join(1197..1777,1861..9034) /gene="pksCT" /codon_start=1 /product="**citrinin polyketide synthase**" /protein_id="BAD44749.1"	2780	4629	92	0	86.63[a]	9269	AB167465.1

Table S6. Trinity-predicted CIT biosynthetic genes BLASTn alignment scores with highly similar sequences from the whole genome of *P verrucosum* BFE808.

Trinity genes	Max Score	Total Score	Query Cover	E value	Per. ident	Acc. Len	Location on *P verrucosum* BFE808 genome (LAKW02000010.1)
g06943	1585	1585	97%	0.0	100	23990	Penicillium verrucosum strain BFE808 Contig10, whole genome shotgun sequence
g06944	1365	3282	100%	0.0	100	23990	Penicillium verrucosum strain BFE808 Contig10, whole genome shotgun sequence
g06947	2623	3556	100%	0.0	100	23990	Penicillium verrucosum strain BFE808 Contig10, whole genome shotgun sequence
g06948	1452	1452	100%	0.0	100	23990	Penicillium verrucosum strain BFE808 Contig10, whole genome shotgun sequence
g03740	1338	2779	100%	0.0	100	23990	Penicillium verrucosum strain BFE808 Contig10, whole genome shotgun sequence
g03741-g03742	7873	8445	100%	0.0	99.98	23990	Penicillium verrucosum strain BFE808 Contig10, whole genome shotgun sequence

Table S7. Proteins sharing > 80% similarity with OTA-PKS from *P. verrucosum* 242724.

Description	Scientific Name	Max Score	Total Score	Query Cover	E value	Per. ident	Acc. Len	Accession
uncharacterized protein N7516_010768 [Penicillium verrucosum]	Penicillium verrucosum	4582	4582	99%	0	99.24	2560	XP_057070707.1
ochratoxin A polyketide synthase [Penicillium verrucosum]	Penicillium verrucosum	4570	4570	99%	0	99.24	2541	AXR84710.1
polyketide synthase [Penicillium nordicum]	Penicillium nordicum	4470	4470	99%	0	97.07	2538	AUS29495.1
ochratoxin A polyketide synthase [Penicillium nordicum]	Penicillium nordicum	4451	4451	99%	0	96.48	2542	AXR84711.1
polyketide synthase [Aspergillus affinis]	Aspergillus affinis	4095	4095	99%	0	89.36	2543	XP_052951117.1
polyketide synthase [Aspergillus affinis]	Aspergillus affinis	4084	4084	99%	0	89.11	2551	UVI58148.1
polyketide synthase [Aspergillus cretensis]	Aspergillus cretensis	4039	4039	99%	0	87.15	2551	UVI58152.1
polyketide synthase [Aspergillus pulvericola]	Aspergillus pulvericola	3991	3991	99%	0	86	2533	UVI57908.1
polyketide synthase [Aspergillus elegans]	Aspergillus elegans	3850	3850	99%	0	82.68	2536	UVI57903.1
uncharacterized protein P170DRAFT_380337 [Aspergillus steynii IBT 23096]	Aspergillus steynii IBT 23096	3841	3841	99%	0	82.62	2532	XP_024705325.1
polyketide synthase [Aspergillus steynii]	Aspergillus steynii	3840	3840	99%	0	82.47	2552	AHZ61902.1

Table S8. Proteins sharing > 80% similarity with CIT-PKS from *P. verrucosum* DAOMC 242724.

Description	Scientific Name	Max Score	Total Score	Query Cover	E value	Per. ident	Acc. Len	Accession
Male sterility NAD-binding [Penicillium verrucosum]	Penicillium verrucosum	2845	3019	98%	0	99.86	2583	XP_057070794.1
Male sterility, NAD-binding [Penicillium expansum]	Penicillium expansum	2734	2898	98%	0	95.5	2583	XP_016601791.1
Male sterility, NAD-binding [Penicillium expansum]	Penicillium expansum	2734	2898	98%	0	95.5	2583	KGO36753.1
citrinine polyketide synthase [Penicillium expansum]	Penicillium expansum	2732	2899	98%	0	95.5	2583	AKN50594.1
Male sterility, NAD-binding [Penicillium expansum]	Penicillium expansum	2732	2897	98%	0	95.5	2583	KGO69364.1
Male sterility NAD-binding [Penicillium expansum]	Penicillium expansum	2730	2895	98%	0	95.36	2583	KAJ5490118.1
Type I Iterative PKS [Penicillium camemberti]	Penicillium camemberti	2729	2893	98%	0	95.43	2583	UPX44771.1
Male sterility NAD-binding [Penicillium odoratum]	Penicillium odoratum	2593	2751	98%	0	89.87	2583	XP_056993194.1
hypothetical protein MAP00_006380 [Monascus purpureus]	Monascus purpureus	2500	2500	93%	0	85.81	1849	BDD61330.1
citrinin polyketide synthase [Monascus purpureus]	Monascus purpureus	2498	2601	97%	0	85.81	2584	BAD44749.1
Citrinin polyketide synthase; Short=CitS; AltName: Full=Non-reducing polyketide synthase citS [Monascus ruber]	Monascus ruber	2498	2638	98%	0	85.81	2593	A0A161CEU9.1

Table S9. OTA genes identified by Trinity Software from RNASeq library and on the genome of BFE808.

**OTA genes**						
**Gene names**		**Genome**	**Gene ID**	**TRINITY CDS**	**BFE808 Scaffold**	POS
AUS29495.1 polyketide synthase		P.nordicum	g09503	TRINITY_DN2062_c0_g2_i4_orf56	ENA|LAKW02001000|LAKW02001000.1	16495
AUS29494.1 non-ribosomal peptide synthetase		P.nordicum	g07679	TRINITY_DN687_c2_g1_i12_orf13	ENA|LAKW02001000|LAKW02001000.1	9192
AUS29493.1 cytochrome P450 monooxygenase		P.nordicum	g07680	TRINITY_DN687_c2_g1_i12_orf17	ENA|LAKW02001000|LAKW02001000.1	6589
AUS29492.1 bZIP transcription factor		P.nordicum	g07678	TRINITY_DN687_c0_g1_i2_orf4	ENA|LAKW02001000|LAKW02001000.1	933
AUS29491.1 halogenase		P.nordicum	g07677	TRINITY_DN687_c1_g1_i1_orf1	ENA|LAKW02001000|LAKW02001000.1	4985
Snoal	JANFQR010000133.1:52439-52791 Penicillium verrucosum strain IFST Pver2 NODE_133_length_67885_cov_285.531642, whole genome shotgun sequence	PvIFST	g07681	TRINITY_DN687_c2_g1_i12_orf1	ENA|LAKW02001000|LAKW02001000.1	15019

Table S10. CIT genes identified by Trinity Software from RNASeq library and on the genome of BFE808.

**CIT genes**		**Genome**	**Gene ID**	**TRINITY CDS**	**BFE808 Scaffold**
ACA34720.1 CtnD		Monascus	g06943	TRINITY_DN1403_c0_g1_i109_orf21	ENA|LAKW02000010|LAKW02000010.1
ACA34721.1 CtnE		Monascus	g06947	TRINITY_DN1403_c0_g1_i109_orf22	ENA|LAKW02000010|LAKW02000010.1
citS_PKS	gi|1418047425|gb|LAKW02000010.1|: 13960-21033 ORF4 CDS CIT PKS PvBFE808		g03741	TRINITY_DN1247_c1_g1_i2_orf20	ENA|LAKW02000010|LAKW02000010.1
			g03742	TRINITY_DN1247_c3_g1_i1_orf6	ENA|LAKW02000010|LAKW02000010.1
CtnB	JANFQS010000410.1:10613-11386 Penicillium verrucosum strain IFST Pver1 NODE_410_length_22563_cov_209.018997, whole genome shotgun sequence	BFE808	g06948	TRINITY_DN1403_c0_g1_i109_orf14	ENA|LAKW02000010|LAKW02000010.1
CtnC	JANFQR010000405.1:398-2069 Penicillium verrucosum strain IFST Pver2 NODE_405_length_22563_cov_287.358100, whole genome shotgun sequence	PvIFST	g03740	TRINITY_DN1247_c0_g1_i1_orf4	ENA|LAKW02000010|LAKW02000010.1
CtnR	JANFQR010000405.1:13827-15500 Penicillium verrucosum strain IFST Pver2 NODE_405_length_22563_cov_287.358100, whole genome shotgun sequence	PvBFE808	g06944	TRINITY_DN1403_c0_g1_i16_orf86	ENA|LAKW02000010|LAKW02000010.1

OTA-PKS (otaA) gene sequence:
>g09503_otaA_PKS
ATGTCCATTGTCAGCGGCGTCAACCTCATCGAACATCCCGCCGCAACCCTCTATCTTTCGAACCTCGGTGTTCTCTCTCCCGATGGACGAAGTTGGAGTTTTGACGCAAGAGCAAATGG
ATACGGGCGTGGCGAGGGTCTCGGGACGGTGATCATCAAGCCTCTATACGCCGCTCTACGCGACGGGAACCGCGTTCGAGCCGTGATCCGAGCCTCCGGTTCCAACCAAGATGGAA
GAACGCCAGGGATCACTGTTCCTAGCGTGGCTGCGCAAGCGCAACTTATTCGCAATGTTTACAAATCTGCTGATCTTGACCCCTCGCAAACAGGGTATGTCGAGGCCCACGGAACCGG
AACCCAGGTTGGCGACCCCTTGGAGGTACAGGCTATTGTGTCAGCCCTGGCTGACCAACCTCGCGACACGCCGCTTTATGTCGGGTCTGTTAAATCAGTCGTCGGTCATCTGGAAGGA
GGAGCTGGATTGGCGGGTCTCATTTCTGCTACTCTGGCGGTGGAATCGAAAACCGTTCCGCCTGTCGCTGGCCTGAAGACATTGAACCCTCGAATCGTGCAGCGAGATGATCTAAAGT
TTGCACGACAGGCTACGCCGTGGCCTAGAGACAATGTTCGCCGCGCGTCTATTAACTCATTTGGGTTCGGCGGTACGAATGCACATGTTGTACTCGACGATGTTGAGGGGTTTTTAAA
TGAGTTTCTTGGCTCCCATTTGACGGGAACATTGCAGGTGATAGGCCCTTCATCGACGACCTCAAGACAAGTGCATGTCCATGCAATCAATGGCATCAAGCCTGTCTCCAATGGCCATG
TGAATGGTGTGAATGGTGCAAATGGCGTTTCTCACTCTAGTTGGATTCCAGAGAACCGAGTTTTCATTCTGTCAGCATTTGATGAAGCTGGTTTGGACCGGAATGCCGCGTCTATGAT
ATCCCATCTCGGATCGCTAAATTTCCAAGGAGACTCAGATATGGAAGAAGGTTACATTAATGATCTGTGTCACACGATAAACGAGAAGCGTACACGATTTGACTGGCGCAGCTACCAT
GTCGCTGACACTGTCGAGAACCTACAAAAGTCGCTTCAGAATGTCCGTCCACTTCGTCGGTCAGCAGCACCAAAAACAATCCGGTTCGTTTTCACGGGCCAGGGCGCTAATTGGGCTG
GTATGGCTCAAGACCTGATGGTGTATCCGCTATTTCAAAGACGTATCCATGAAGCTGCGTTGTTTCTGAGGAACATTGGATGCCAGTGGGATCTTTACGACCGGATAACCTCACAACA
CGGTGACCTCAATGAGCCTACCTTTGCGCAGTCATCCTGTGTTGCCGTGCAGATTGCGTTGGTGGACCTGCTGCATAGTTGGAAGATCACGCCATCAACGGTGGTGGGTCATTCTTCT
GGAGAAATTGCAGCGGCTTACTGCGCAGGGAAGATCTCACGCCAGACAGGATGGAAGGTTGCCTACTGTCGAGGTCAGGTTTGTGCAAAGCAGACACACGAAGATGGCTGCATGCT
TGCTGCGGCTATGCCTGCAGAAGAACTGGAACGACTGGTAGCCCGCGTAAATAAAGGTCTATCATCTGCGGTTCAGATCGGATGTTACAACAGTCCCAAGAACTTGACCCTGACAGG
CACACATGAGGGAGTCCTTCGTGTCAAAGGAGAATTAGACGACGCGGGTGTGCTAAATCGCTTGCTTCCCGTCAAGGTAGCATATCATTCCGTCTTCATGCGCGAAGTCGCTCCAGAA
TATCTTGAGCTTCTGGGAGATCTGGATTTCGGTGAGAAAATCAACCAGGAAGCGGGTGTCACCATGGTATCCTCAGTTACCGGTCGACCTGCACTTGCCGGTGAGGTTGAGAATCCTT
CATACTGGGTCGACAATTTGGTTTCGCCTGTCCGTTTCTCCACTGCGCTCCTCGCATCAATGAGAAATCCGAACACGTCCGGCTCCCAAGAAGATGCACTTATTGAAATCGGGCCGCAC
TCGACCCTTCGCAGCGCCATAAACGAGACGCTTGCAAATCATCCTGAGCTGCAGCCATTCCATTACGGAAGTCTTCTCAAGCGATATAAGACCGACGGAACAACAATTCTGCACACCTT
TGGCATGCTGGCTTGTTATGGCCATGAGATCAACTGTGTTGCCCTTAATGATCGCCGATCGGGAACCAAGAAAGTTGCCAAAATCCTTAATGATCTCCCGCCATACGAATTTGACCACT
CAAGATCCGTCCGTGGCCAGACACGAAGAATAGAACACATTAAATTCCCGGCCTACAAACGTCACGAGCTTCTCGGAATGCCAGTTGAAGACACAAATACATTCGAGCAGCGATGGA
GGAACATATTGAGACCGGACGATATCACATGGCTGCGCATGAACAGAATGAAAGGAAATATTCATTTTCCCGGCGTCGCCTATCTTTTGATGGCGATGGAAGCCGCCGTTCAGCGGG
CTGGGGAGTCAGAGTCGATCACCGGCGTCAGGTTAGGCAATGTGTCTATGTTGGCTCCTCTGCCTGTTTCGGACACCCCAGAAGGGGTCGAAATCATCTCTTCAATCTACCCAATGAA
CGCTTCGGCCAATGGCAGAGATGACTGGTGCACCTTCAGAATCATCTCGCATGATGCTGCTGACAATGCTTGGATCGAGCACTGCGTTGGCTCAGTTCGTATAGAGACGAGTGAACA
GAAAGCATCCCCCTGTCCCGTGAAGAAGGAATCTCTATTAACTTGCGAGAAAAGCGTCGACATCGACCAAATGTATCAACGCTTTGGCGCGGCGGGTATGGATTTTGGAGAATTCTTG
AGGAACATCCAAGATATGAAACTTTCCGCCGACCATAAAGCCTGCACAGCAACTATCGCGACGCCAGATATCCCAAAGACAGCTCATGATCGATATATTATTCACCCGTGCACGTTTGA
AAGCATCTTGCACGTACTTTTATATCTTTGCGAAGCCACAAGTAGTCCCATGGTAACGAACTACATTGAAGAGGTTTTTATATCATTTCCATCCTACCAAGCAGACCCTAAGTTCCAGGC
CTTAGCTGCAACGAACAGGACATCTTCCACAACTTGGAAGTCAGACGTGTTAATCACTGCGGAAGATGGAAACCAGGAAATATCAATCAAAGGCTTGGATTTGGTGCAACTTCCTCCG
AGTGCCACCGAATCGTCGGAAGCTGAGTCGTTTTATGTTGTGAAATGGAAGCCAGATGTTCACCTTGTTGCGTCGGTACAAGCTCTACGGGATGCAGCTCCCGTTGAGGTACATCAGC
GTCTTCCGAATTTCGACGATCATGAGGGATTCCAACTCGCTTCGGCGGTCTTTCTGCTTGACACCATGGACTACGTTGAGCGAGAAGGATTGCCATCTCTTCCAGACCATCACAAAGCT
TTCATGGACTGGATGCAGCGGGAATGTGAACTCATCGCGGAATCATCAGTTCCTTATGTTGACAGAGCGCTGCTTGAAGGCATTCGCGAGAGTCCATCTCGACGCAAGGACCTGTTCG
ATCGAGTAGCTAGCAAAAGCGCACGAGGTGAACTTCTCGTACGCGTTGGCCTGCAGATGATTCCCATCTTAGAGCAAAAGCTCGATTGCTTGGACGTGATGTTTGGGGAAGATGACC
TCATGGATCGAACGTACGAGGAAGGTCTTCCCGGTAACATCGCCTCTACGGTCGCTGGCTACGTGCATTGCCTTGCTCATAGTCGTACGGATCTCAAGGTCCTGGAAGTCGGCGCTGG
TACTGGCAGTGCCACGAAGGTAATTCTGGACAGCTTAATGCCAACAGCTCGACTGGATGGAGGAGGCCTTGTCTCGTCTGTCTCTACGTACCATTTCACAGACATATCAGCAGCCTTCT
TTGAGAATGCGATCCAACGGTTCCCGGACTGGGCGGGCATTCTCCGTCCACATGTACTGAACATTGAAAAGGATCCGGCGGCCCAGGGGTTTGAACTTGGATCTTATGATCTAGTCAT
TGCCACACATGTCCTTCATGCAACTGCCGACCTCGCCGTATGTTTGAACAATGTCAAGAAGCTACTCAAAGATGGTGGTGACCTCATTTTGATCGAAAATATCAAACCCGAACTATTGT

GCTCGCCCCTTGGATTCGGCTTGCTCCCAGGATGGTGGCGCAGTGTTGAGCCCTACCGGCAGTTGAATCCTTTGATCAACGAGGATGGATGGCTTGCGGAACTACATAATGCAGGTCT
TCAACCTCGGCTACTAATCAAAGACACAGACGACCAGGTCAACGAAATTTCTGCCTTCGTTGCAGCAAACGTACCATCAGGCCTTCCCACGAAATGTCTCTTCAGCCTAATCTACTCATC
CAACTATCCTGGGCAATATGACCTAGCTTGCAAGATCGCGGACGGGATGACATCGGAAGCACGCACTATCATTCTAGTTGACCTGATCGATGTTTCGATTCATCACACCGACACGATT
GGAATTATCTTACTCGGCTACCAGGGCTTAGACCTCTCTAAGCTCTCAAGCGCAGAGTTTGAGAAAGTCCAGTACATCCTTGGCTCCTTCACCAACCTCCTCTGGGTTTCCCGTGATGTT
GATGAAACACCCAAGTCTGCCATGGCCACCGGGCTTGTTCGCACAGCTCGCTGGGAACGGGATCATGACTGCATCAACTTCCTCATGCTCGAAATCAAGCAACGGCCTGGCGATATGC
AAGGAGTCCTCTCCAATATTACTCGGGTATGCGACCACGCTTTCACATCAGAGGCCCTTGTGCCGCGCAACGCAGAATACCGTCTACAAGACGGGATTCTGCTCACCAACCGTTTGTTC
CCGGCCCCAGGGATCAATGAGTGTATCGATTCCGGGTCTAGGTCACGGTCCAAACTAGTTGAGCTCGGACATGTCACACACCCGATCACGCTCACCTCGGTTGGCCCTCATCAACCGA
ACGGTTTCCACTTCATTGAGGATGAAGACTATAAACGGCCTCTTCATCCACATGAGGTGCAAATTGAAGTCCGCGCTGTAGGTATCGATGAGGAAGATGTTGACAAATTCAGCCGCTC
CATTCCAGGTGAGAGCCTTGGCTGGCAGGGCGCAGGCATCGTTGTCGGTCTTGGCGCCGCCGTACAAGGTCTCCAGATTGGCGATGCTGCCATGGCGTTGCGCACCTCGAGCGGAG
CCTTCCAAACATTCTTTCGCGTTCATTCTGCTGCTGTCGTGAAAGTACCAGCCGGCACCAGCTTCGCCGAAGCCGCTGCGCTCCCAGTCACATTCTCCACTGCATATCACTGTCTCGCCA
ATGTTGCCAGGATACAGAAAGAAGACAAAGTCCTCATTCATCAAGCGTCTGGGGGTATTGGGCTAGCGGCGGTGCAAATTGCTCAATCTCTTGAAGCCACTATCTATTGCACAGTTTC
AGACGACTCCCAGAGACAACGTATACTCGATATGGGAATTCCTTCTACCCAGGTGTTTGGCACAGAATCGTGGAAGACGGAACATTGTGCCGTCGCAAACAGTGACAGAGTCGATGT
CATTCTCAACCTCAGCCCACGTGGAATTGAAAGCAAAGACCTGAATTGTCTCTCCTCTTTTGGACACCTGATAGATCTGCACGGGCACGGTATTCTCGGTCCTGTATCCGCTTCAACATC
GAACAGGACCTACTCTATTGTTGATATCCGTTCTATGGCCCTTCAAAATCCGAAGGCGATCCAGGACACACTCCAAAGTTTAGCAACACTTCTGGCGCAGCAGAAGGTCCGGCCTCTTT
CGCCAACTAAGTTCGGATTTTCCGGTCTTTCGAATGTCCTTTCAGAGATCCGGCAAGGGATCCCCGGGCCTTGGGTTCTTGAGCCACGACCGACGGATTTGATACCTGTCGTTATCAAC
CCACTAGGAGGACACACCTTTGACCCGAACGCGTCATATGTCCTTGTGGGCGGCTTCGGTGGTATAGGCCGGAGCGTCGCTCGATGGATGGTAACTCGTGGCGCGAAGAACTTCATT
TTCGTCTCACGATCAGGCGCCAGCAGTGATCCCGCCAAGCAACTATACACGGAGCTTCTGGCTTCTGGCTGTGGCGTGTCTGATCTATGTTGCGATATAACCAACAAAGAAGCTGTGG
TTAAGGCGATCAGCAGCTGCCAGACAACCATGCCTCCGATCAAGGGATGCATGCAATGCTCCATGGTCCTTGAGGACTCCATGCTCAGCAACATGACTCACACACAGTTCCTCTCCGC
CATCACCCCCAAGGTGCAAGGCACAATTAACCTCGCCTCTGCGCTCTCCAGTTCCCCACTCGACTTCTTGATCTTCCTGTCCTCATCAGCCGGCATAATAGGCAACCGCGGCCAAGCCAA
CTACTCAGCTGCCAACGCCTTCCTAGACGCTTTCGCCGCCTCCCTCGTCCAGAAAGGATACCCCGCCACCTCGATCAGTCTCGGCAGCGTCCTCTCCGTCGGCTGGGTCGCCGAAAACC
AAGACCGCCTCCCAATCGCCCTCTCCTACGGCGCTATCCCCGAATCCCTCCTCCTCTCCATCCTGGAATACCACATGGATCCACGTTGGTGCGCTGCCCAAAGCCCAGACACATGCCATA
CCGTCGCGGGAATTCGCTCTGCGAAAGACTTCCATCGTCAGTCAATCCCATTGCCGGGCTTCATGGCCTATCCGCTGTTCTCGCCGCTCCGTGCAATAGCCAATTCCTCTGGTGCTGAG
AAAGAGGAGGTTGAGGTTCCGGTAACACAGGCGCTTAGATCTGCGCGCTCCACACCAGCAGCGGTAGAGGTTGTTACGAGGGCGATTGTTATGAAGTTAGCGCGTGTGATGGCAAT
CTCGGCGAAGGAGATTGATCCGGCGAGGACGTTGGCTTCGTACGGGGTTGATTCGTTGGTGACGGTTGATTTGAAAGCGTGGTTTAAGAGAGATGTCGGGGTTAATATTACGTCGG
CGGATTTGTTAGGGGATGTTTCTATGCAGGGGTTGGCGGAGAAGGTTGCTGGGACAAGTGAGTTTTTGTCGCTTCGGGAGTAG

>g07679_otaB_NRPS

ATGGAGTCCCGAACCAGCTATCAGTTTCCCGGTCTAACAGATGGGCGCACTGCCAAGGACGAAATGGCATCTATTCCGCTCAAAGGTGTCAAGTACTCCGATCTCAAAAGCGATGATG
AGGATTCGGCAAAGCTGCTTTACATCGCCTGGGCTATTTTGTTATTTGTGTATGAGGCGGAAGCCACCGAATTCGAGCTGATTCGCAATCGCAAAAGATCAACCCTTAGGACGGCGAT
TAACCCCGATAAACCGTGGCAAGATATTCGTTTCCAGTACCAAGATAGTGAGGAGACAGGTGAAATCACTTCCGGTGTCTGTATCTTTACAGAAAGAGATGTCGAAATTCCAGCTGAA
CTGCAATCAGCGTTGGTAATACAATGCTCGGACGAATTTACAGCCTCTCTGGTGTATCGCTCGCATATTGTTTCCGATACACATGCACTTAACATCGCCACCACCCTCGAGAAGATCAC
AGACGGATTACAAGGACCAACTATTCCCATAAGCGAGATGGATGTCCTCAGCGAGCACAACTTCAATCAGATATCAAAGTTCAACACTAGTCCGCGTAATGTCGAGGAGCACTATCTA
TACAGTATCATTGAGAAGCAAACACTCGACACACCGGACCAAGTTGCTGTCGATGCTTGGGATGCTCGATTAACATACAAAGAGCTCGACCTCTATGCTTCGATTATAGCCAAACGTCT
CAGGGCACTCAATTCCGTGAATACATACGTACCAATGTGTGCAGAGAAGTCCGCCTGGGCCGTTGTCGCTATGATGGGAATTTTGAAAGCCGGGTCTGCTTGTTCCCCTCTCGAGGCA
TCTCATCCACGCGATAGATTAGAAAGCATGATTCAATCGTGTGGTGCCAGAACCGTTGTGGTGACGGCTACATACGCGTCACTGTTTCAGATGAAGGGGATTGATGTCGTGGTTGTCT
CTGCCGATTCTCTATCTTCCGCAAAGGATGACCTGAATTTGACCTGGGTTGCCGTCAATCCACGATCGCCAGCCTTCCTCATGTGGACGTCTGGAAGTACAGGTAACCCCAAAGGGGTC
GTCCTGGAACATGCAGCCTTGTACATGAGCATCACCGCATACGCAGAGGCCAGCGAGTTTACAGCGAAAACAAGAACATTCCAATTCACCTCTTTTACATTCACGGTCAGCATATGCG
ATATCTTCGGCACCATGGCCAAAGGAGGGACATTGTGTATGCCGTCCGATGGGCAACGTTTGAACGATCTGACTGGAGCATTGAAAGACTCGGCCGCCTCCTTTTGCTGGCTCACTTC
GACATCCCTTGTCGGACTGGACCCTGATAAACTCCCTGATCTCCGGTGTGTCACTGTGGGTGGCGAATCTCTTTCCCGGGACGTGGTTGTGCGCTGGGCCGGAAAGTGCCAGATCAAT
GTGTCATACGGCACAACCGAAACGTGTGGCTGGTGTTTGCTGAACCCACACCTGACTGCAGAAAGCGATTCCCGAGTTCTCGGGAAGCCTATTATCCCCGCAGCTTGGATATGCCATC
GCGATGACATCGAGAAGCTGGTCCCTGTCGGTGCGGTCGGCGAGCTGCTAATCGAGGGGCCATTTTTAGCGAAGGAATATATGGACGACCCGGAACGTTCTGCAGCGCAATTCATCC
AGCCACCGAGTTGGATGAAGCGATTCCGACCAGGCCAATCAACGAGGCTTTACCGAACGAACGACCTGGTCAGGTATACCTCGGATGGATCTATCAGGTTTGTAGGCCGGAAGCAG
GCCCATGCGAAAATCCGCGGGAACCGGATTAATCTCATCGACATTGAACATCACGTTCGAAGCGCATTGGGAATATCCGAAGCAGTTGTCGAAGTGGTGCATACGAAAGACGGTGTT
GATATGCTCGCGGCATTTCTATTAGCCTCCTCAGACACGGGCATGAACCAGGGCTCCGAAAGCCCCGTGACCCAGGGGGATGATGCTATCCGTCAGTGTGTTGCCAATGCTTTGCAGG
CCCTGGAGACCGATCTTCCCAGCTACATGATTCCAACTGCCTTCGTACCGTTATCTCGAATCCCACTCACGCGTACCAACAAGACAGATCGTCGATTGTTGTGCGAGGAGGCAGCGGG
CCGGACAAGAGCTGAGCTAGTGCAGCTTTCAGCGCACAGAATTCAAAATGTGGAATCCTCACTCTCAAGGACTGAATACATTATGCAGCAGTTGTGGTCGGAACTGACAGGCCTAGC
GATCCAAAATATCGGCCCCGACGACAGCTTCTTTCACCTAGGAGGGGATTCAGTCATGGCAATTCGGCTGGTTCCGCTGGCTAAGAAGCACGGGCTTGTATTCACTGTCATGGATATG
TTTCGATACCCAAAATTGAGAGATCTGGCAACTTATATCGATGACCGCGGAGAGCATGAATCGACCGAGAAGGAATTTTTGACCTTTGAGGTCGATGACCTCGCCCCAGACGCCGCAA
GGGAGTGTGGGGTTAATATCGATGCGATCGAAGACTTGTATCCTTGCACAGCATTGCAAGAGGGGCTTATGGCACTATCCGCACAAAGAACTGGTGCTTATGTTCTGCAAATGGCCT
GCGATGTACCTTTAATGGCAGATCTGTCCCATCTTCTAACTGCATGGGAGACTGTGCTCGATTCCCTTCCCATACTGCGCACACGAATTGTTCAGCTTGGTCGGGCGGGATTCCATCAA
GTTGTCGTGAAAGAGAAAATAGACTGGCGCCCTGTAACCAGCGAGTCGGAGTGGCGAGACTGGAATCACAAATATCCTATGCAATTAGGATGCCCATTGGCGCGATTCGCTCTCCTT
CAGGTGGACTCCCGACAAACACGCATCCTGATTTCTCTTCATCATTCCATCTTTGATCGCTGGTCATCTGTACTTCTGCTCGAGATGGTTGATACAATTTATCAAGGACGACAGGTGCAG
CGACAGGCGTTTAAAGAGTTCGTTGGATATGTGCACTCCCGTCCAACTGAGTCGAGTGACGCTTTCTGGGAAAATTATCTTGGAGACGCCCAGCACGTGGCCTTCCCTCGCGTGAAAG
ACGCGAATTACCTTCCCAAGCCAAGTGTGGGAAAGAATAAAGTCATCAAGCTCGGGCACTCGAGAAGCAATTTCACGGCGACCACAAAGTTGCGTCTTTGTTGGGCTCTAGTTCTTGC
CCAGCACACAGATAATACCGATGTGGTGTTCGGGGCTGTCTCGACAGGCAGAAGCGCTCCCGTTGAAGGTATAGAGAGCTTAGTTGGTCCGACTTTAGCGACTGTGCCATTCAGAGT
GAGAATAAACCTGGAGACCTCGGTCACCGATGCTCTAGATAGCGTGCAGCGTGAAAGCGCGTCAATTCTCCCCCATGAGCAACGCGGTCTACAGAATATCGCCAAACTGTCCGCAGA
CACAAAAAGTGCTTGCAAGTTCGAAAATATTTTGATCGTTCATGCTCCACATTCAGGCGGGGGGATGAGTCTTCTTGGGGACATGGCTGATGATCAATTGCCGGAGCTTTTCAGCTAC
GCCCTTACATTATCATGTGAAGTGCTCGGAAGAGATCAAATTCATGTCGACGCGTTCTTCGACCCGAGAATCATTGGAGAGAGATACGTTGAGGCCTTACTTTGCCAGCTGGACTATG
TGATGCGACAAATACATGATGTGCCCGACTGCCGACTTGGAGACATCAGCGCTCTTAGTGGCAATGACGAGCTATTGCTAGAATCATGGAGCTCACGATTGACTGAACCGGTTGATTT
GTGCATTCATGACGCTATCCGGGACCGATGTATAGCATCACCTGATGCAGAGGCTGTGTGCTCTTGGGACGGATCGTTTAGTTATGGCACTCTTAAAGTTCTTTCGACAGCACTCGCCA
TTCAGCTCCATCACCGAGGAGTAGGCCCAGAAGTATTCGTCCCCTTGCTTTTCGAGAAGTCAAAATGGACAGTGGTCGCTATACTCGCAGTCCTGAAGGCCGGTGGTGCTTTTCTTCTA
TTAGACGCATCATTCCCCAACGACAGGCTGGAAGCGATTTGCAACCAAGCAAGCGCACAAGTTATTCTCGCCTCAAGAGACCTATCCGAGAGAGCAACAATCCTTGTCCCTGGCAGCC
TAGTGGTGGACGACACGCTAAACTCTAACTCAGCACCTTCAATCATATTACCGACCATCAATCCAGCCAATGCCCTCTACGCAGTGTTCACTTCAGGGTCAACGGGAAAGCCCAAGGG

TGTTGTGATTGAACATTACGCATATGCCTCTGGGGCCAAAGCGCATATTGCAGCTGCATCGATAAAATCGACATCCCGGGTTCTGCAGTTCTCCTCATACGCATTTGATGCCAGCATTA
TTGAACATTTGACAACGCTCATGGCTGGTGGATGCATCTGCATTATTTCAGATAAAGAACGTGACAACTCGTTACCAGAGGCAGTCGCGTCCAGAAACGCCAATTGGACATGGCAGAC
ACCTAGTGTTGTTCGGACAATGAATCCCGGGGACTTTCCTTCACTGAAACACCTTTGTTTGATGGGTGAGGCTATTGGACCCACAGAAATCGAAGAATGGGCCCAACACCTTAAGCTC
ATGCAAGCATATGGGCCGGCGGAGTGCTCAGTATTGGCAACCTTGCAGACTTCGTTGAATCTGAATTCGGATCCTCGGAACATCGGATTTCCAACTGGCTGCACTGGTTGGATTGTGG
ATAAAGATGACAATACCCGCCTAGCGCCTATAGGTGCGGTGGGAGAACTTCTAATTGAAGGCCCTAGTGTGGGAAGGGGCTATCATGGGGACCCGGAGCAGACTGAAACAGCGTTT
GTCGAGTGGCCTGATTGGATACACAGATATCGAGAGTCTGGTTCTCCAAGAAGTTGTCTGTACAAAACAGGCGATTTGGTTCAGTACGCTCCAGAGCTTGACGGCACACTGCTCTATA
TTTCTCGCAAGGACACGCAAGTCAAAATCCGTGGTCAGCGTATGGAGCTTAGCGAAGTGGAGTACCATACTCGTGAAGCCATGAATTCGTCCTGGGACATCGCAGCCGAGGTCGTCC
AGCATGGGACCCGGAAGATTCTTGCTCTTTTCTTCGCTGACAACTTGAAGACTAATGCGCAAGAGAGTTGCTTTGCTATATCCATGAAAGATGAGGTGTGCTCTGAAGTGGAGAAGGT
TAAGCATAAACTCAGCACTCGCTTACCTGCCTTCATGATCCCAACGGCTTGGATCCCAGTGTCCAGAATACCACTTTCTGCTTCAACAAAGACAGATCGGCGTCGAATGCGCCTTCTCGT
GGACGATATTCCTGTGGAGCAATTCAACACTTACATCATCGATGGCAAGCGTCGAAATGGACAGGCCTCAAATAGCTCCACACGTATTGGTGATAAGCCAATGACCGAAGAGGAGAT
TATGCTACATGGCCTCATCTGCGAGGTACTTGAAAGTCAAGGCCACGAGATCGACAGAAATACCGTCACCATGGACGAACAATTTACTAGCATTGGAGGGGACTCGTTGACAGCCTT
GTCTTTAGTTTCAAGGGCACAGAAGATTGGATTTACTTTTACCGCGACGGACGTGATTGCGTGCTCTTTGGGGGAGTTGGCCAGTAAGGGAGTTTGA

>g07680_otaC_Cyp450

ATGGACTCGGATATATCCGCCTCCAAATTACGCTTTCTTCTTTGGTCGTTGTTGGTACCACCCGTCATTTATGTGGTCACTACCGCGACCTACCGGCTTTTCTTTTCTCCGCTTTCACATAT
TCCAGGGCCTAAACTGGCCGCATGCACACGTCTGTACGAGTTCTATTACGATGTTATCCTACACGGTAGGTATACCTTCAAGATTGCTGAGCTGCACAAGAAATACGGCCCCATAATCC
GCATCAGCCCTGGGGAAGTGCACATCAATGACCCCGACTATTACGAAACTCTTTATTCAATTAATGGGTCGCGCAACAAGGACTCATGGTTCATCGAGTCGTTCGATGTCGCCGAGTC
TGCCTTCGCCACGCTGGATCATCGTTTGCACAGACCCCGTCGGGCCCTGATCGCTCCGTACTTTACCAAAGCCCGTGTGCAGCGTGTGCAGTCGCTCATTCATGATAAACTGCAGAAGC
TCACTACTCGATTCAATGAGGTTGCGCACTCAGGAAAGCCACTCAAAGTGGACGTCGCTTTCAACTGCTTCACCGCCGACGTGATCACATCTTATACCAGCTTTCGCGCGTTCAACTATC
TCGATGATCCTCATATGATGCCCATATGGAGCGAGACAGTCCGCAACCTTGTCGAAATTGGAATGCTCGCCCGGCATCTACCTGGCTTCTTCCCGCTTCTGGTGAGTATGGGGATGAA
GTGGATTCAGCGAATTTATCCGAAATTGCTTCCTGTCATCGCATTCCGGATGAAATGTGCGGAAGAGGTAAAGGCCATGTGGGTGAACGAAAAAGAATCAAAAGAAGAATTTGAAAA
GAACAGACTTTCTCAGGAGCCCGCGCTGTTCCAGGAAATGGTCGCCAAAGCCCCGAACACCCCTGATATCACGGAGACTAGAGTTCTTCATGAGTATATTACTATCGTGGCAGCGGGA
ACAGAGACCACGGCGCATACGATGACGGTCTGCACCTTCCACGTCCTCAATGATAAGGAGGTTCTTCAAAAATTACGTGCGGAGTTGGAAGAAACCTTTCCGGAGAAGAAGGAAATG
GACTTACAGACGCTTGAACAACTCCCCTACCTGACTGGAGTCATTTACGAAGCACTCCGACTATCATATGGCCTTTCCCACCGTCTGCAGAGAATATGCCCAACCGATCCACTGCAGTA
TAATGACGTTGTCATTCCACCCAATACATCGATCGGAATGTCCGCGGCCCTTATCCATCACGATGAGTCTATCTTCCCTAACTCGCACGAGTTCATCCCGGAGAGATGGACAAACCTTG
AAGAGAGGAAAAGGTTGAACAAATACCTGGTTTCCTTCAGCAAGGGTGCCCGCCAATGTATCGGAATGAATTTGGCATTTGCGGAGCTTTATATGGCTGTCGCTACGGTGTTCCGCAC
ATTCGATATGAAGTTGCATGAGACTACGGTGGACGACGTCCGACTACACAGCGACATGATGCTGCCACATCCCAGGTCGGGTAGTAAGGGCGTGAGAGTAACGATTGACCGTATCCA
GTAA

>g07677_otaD_Halogenase
ATGTCAATTCCAACTAAAGCGACGGCGCTCGTAATCGGCGGTGGTCCGGGAGGCTCCTACGCGGCATCAGCCCTGGCGCGGGAAGGAGTCGACACGGTGCTGCTAGAAGCGGATGT
GTTTCCTAGATATCATATCGGTGAGAGTCTCGTCGCCTCAATCCGACCGCTTTTGAAGTTCATCGACTTGGATGACACCTTCGTGAACTATGGCTTCGTGAGAAAGAACGGAGCAGCAT
TCAAACTGAATAACCAGAAAGAAGCTTACACGGACTTCATTCTCGAACCTGGCGCTGGAACCTACGCGTGGAACGTCATCCGATCAGAGTGCGATGAGCTGATGTTCAAACATGCCGC
GAAGTCTGGAGCGAAGACATTCGACGGCGTCAAAGTCACGTCGATCGAGTTCATTCCAGATGAAAGCAACGTCGAAAGCCCCGGTCGACCGGTGTCCGCTAGCTGGAAAACCAAGG
ACGGTCGCACTGGGATTATTGATTTCCAATACCTCGTCGATGCAAGTGGTCGAGCCGGTATCACCAGCACCAAGTATCTGAAAAACCGAACTTTCAACAATTACCTTAAGAACGTCGCT
AGCTGGGGCTATTGGCGCGGTGCGACACCATATGGCGTGGGAACTCCGGTAGAGGGCCAGCCTTACTTTGAGGCCCTTCAGGATGGCAGCGGCTGGGTGTGGTTTATCCCTCTGCAC
AATGGCACGACTTCCGTCGGTGTGGTCATGAACCAGGAGATGGCTACAAAGAAGAAAAAGCTATCATCCGTGACCTCCAGTCAAGCTTTTTATCTAGAGTCGCTCGAAGGAGCGCGC
GGGATCTCTCGTCTCCTCGAACCTTCCACGCTCGAAGGCGACATCAAACAGGCGTCGGATTGGTCCTATAATGCCTCTTCATACGGCAGCTCCCACCTGCGCATAGTCGGGGATGCCG
GTGCATTCATCGATCCGTACTTCTCATCGGGAGTTCATCTGGCTCTTTCGAGCGGATTGTCTGCCGCAACATCCATTTGCGCGTCTCTGCGCGGCGATTGCGAAGAAGAAGCTGCGTG
GAAGTGGCATTCCCAGGGCGTGGCTAATAGGTACGGTCGCTTTTTGCTGGTAGTGCTCGGGGCGACGAAGCAGATTCGGGCGAGAGATACCCCGGTCATGAACAAGGATGGTGACG
AGGGGTTCGACGATGCGTTTACCGTAATCAGACCCGTTATTCAAGGAATCGCCGATGTCCCTGGTAGAACCACCCATAGAGAGATCCTTGATGCCGTTGCCTTCAGCACGAACGTCGT
AGGGCCCAATATTAAAGGCACGGAGCAGGCCTGGGTCGAGAAAGACCATGGTGCTTTGTCCTGCGACGAAGAGGAGGTTGGCAGGGTCATGAATAATCTGGCCAAGGCGTACAAA
GCACAGGATGTCTATGAAGGGCTCACAGCAAGACTTGAGAGAGGAGCGCTGGGATTGCAGGTTGCTAATTAG

>g07678_otaR1_bZIP_transcription_factor
ATGGACGATATTCGCTTGTCCGCAGACCTGCCGGTGGACTGGACTGATATGCTGTCGCAATTGAATGCCAGCGGAGATGTTTCGGGCGCTCAGCCTAGCTCGATGGCCCCGAGAGGA
GGATTCGAATCAAATGGTGTCTCTGTGACACCTATAGCGCGAAACAATGATCACTACCCATATCAGCCCTCCATGCTAGAGTTGAACAACCTTGATCGAATTCTTGCGTTACCATCTTCG
AGCGCCAATAATGGCACTCCAGGTCCTCATACGCCTTTTTCCGAACCTCATGATCTATTCACTCTCCTCCCAAACACAACGCCTTCTACGGCAAATCAAGAAGAGACCGATGCAGCGGC
GCAGCCAATTTCGGCCGGAGCGGATTCTCCTTCCGGCGAGACGAGACCCACGTTTGAAAAAAGCGCCGAGGTGAAACGACTGCGGCAGAAATATCACGAAAAGTACAAAGAGCGCA
ATCGTTTGGCTGCAGGAAAGTCGCGACAGAAACAGGTCGATTTAATCGCTCTCCTCGAAGCGGAGCGGCGAGATGAAGAGCGCCGAAGACGGGTTCTTGAGGATGAAATTCAGAAA
ATAAAGAAAGATCTATTTGCTATCAAGCAAGAGCTTCATCATATTCGGGTGTTCAATTGTATGGGCATGATGTCGCAGGGCGCTCGTCTTCAAACTCTGGGCCTTCTGGCACAAGATAT
CTTTCGGTAA

>g07681_otaY_SnoaL_Cyclase
ATGGATCTCAAATCCCGTGCTCAACTGTTTCTGAACACAATCGTGAACAAGCGGGAAACATCCTCCATTGCCTCCATCCTCCATCCAGACATCGAGTTACGACACGATGACCTCCCTCCA
ATGTCAAAGCCAGAGTTTATCGACTTCTGGCCCCAGCTGCTCGCGCAAAGTCCGGATTTCACGGTGGAAATCCTACGAGTGATCACTGAAGGCTTACACGTGTGGGTCTATTCTCGGG
TCAAGGGACGACTTGGTGGAGGACTACTGGATGATGTTCATATTTTGGATTTTGACGAGAATGGATTGTTGATTAGAAGCAAGGGAATGCAGAGGGAAATCCAAGTACATCCCGAG
GACGAGAAGAAAGTTGAAGGAAAGTTGGGCTAG

>g03741_pksCT_citS

ATGAGAAAACCGGTTTTCTTCGCCCATGCAGTCAGGAGATTATCAGACAAATTCCCTGCTGCCACTTGGTTAGAGGCCGGTTCAAACTCCACCATCACAGCCATGGCAAGTCGGGCTC
TGGGTACTTCAAACTCCTTTTTCCAGGCTGTGAATATTACCAGCGAGGGTGCATTCCAATTTCTGTCGGAAACGACTATAAAGCTCTGGAGGGAAGGTCAGAAAGTCAGCTTCTGGGC
TCACCACCGCCTGCAGACTGCAATGTACACTCCAGTCCTTTTGCCACCGTACCAATTCGAGAAGTCAACACACTGGATGGATCTGAAGGTGCTTCCAAAGCCCGAAGTTTCTGCCAAGG
TGACGGAGCAGCCCGCACTTATCGAGGCACCGAAGGGCCTGACGACTTTGGTCGGTTATCAAGATGCATCCCAGAGGTCTGTGCAATTCAGAGTCAATGTCACGACAGATAAATTCA
ACCGTCTCCTTTCTGGTCATATCATGGCAAATACATCTGCTGTTTGTCCCGGTATGTTCCAGGTTGAGGTAGCTCTTGATGCTCTTATGAGCCTTCGACCAGAGTTCCAGAACCGTAGCT
TTATTCCAGAGCTACATGGTCTAAGACATTATCAGCCCCTTGTCAAAGACGACTCCCGGGCAGTCTGGATCGAAGCGCAATCCTTCGATGCGGAAGGTCTGGTCTGGAATTGGAAGCT
GACTGCAACTGACGATAAGGGAAGTGGCTTTATAACCCACACTTCAGGAACAATCATATTCCAAGCGGCAGACAGTGTGCAAGTCAAGACTGAGTTCGAGAAACTTAGACGTCTAAT
CGGCCGAAAGCGGTGCTTGCAACTCCTTGACGGCAATGTGGCAGACGACATCCTGCAGGGCCGCAACATCTACCGCGCTTTTTCGGAAGTGATCGACTACAAGGAAATATATCGTCA
TGTTACAAAGATTGCTGGCAGCGAGAACGAGTCCGCAGGAAGGATCACGAAGAAATATGACGGAGAGACCTGGCTCGACACCGTCTTGACGGACTGCTTCTGCCAAGTGGCAGGTA
TCTTTGTGAACCTCATGACTACCAAGATCGACTTGTCTGAGAGAGGCATTTTCATTTGCGATGGAATTGATCGGTGGATGCGAGCACCGAACGCAGATGCAAACGACGCTCCTTCACA
CGTCTATGAGGTATTTGCTTTGCACCATTGCGAATCAGAGTCCAAGTACCTCAGCGATGTTTTTGCTTTCGATGCTCGTGATGGGTCACTTGTTGAAGTTGCTCTTGGTATTAGTTACCA
AAAGGTTCCAATCTCCGGAATACGCAGGGTACTGTCGAAGGCTATGCCAGTTGGTATTCAGCCACAAGTTCCTACTGCCCCTGTTGCAGTGCCTGCCCCCAAGATATTCAGCCATACTC
CAGTCGCAGCCCCACCATTGGTGAATGGCTCGTCTAGTGCGGTGAACGGTACACCCCCGACAAAGAAAGCGCCAAATGCAGCCAGTGTAGACATCACTAAAAAGATGCGGGAGATC
ATATGCAATTTATCTGGTCTTGAACCAGAAGAAGTCAGAGACAACTCTGATTTGGTCGAGCTTGGAATCGATTCATTGATGAGCATGGAACTAGGCCGAGAGATCGATTTGGCTTTTA
AGACCACTGTCGACGTCACTCAGTTGATCGATGTAACTGACTTCCGCAGTCTTGTGGAGTGCATGCAAAAGATTCTGGGCATTGAAAAAGCCGAAAAGAATAAAGAAGCTGCTACTA
ATGGAAACACACATCATCTCAACGGCACAAATGGTCTCGTCAATTGCAGCGGTCTCTCGTCTCCTGGGGAAGGGGGTTCCCTCCTCTCAGAATCGGCAATCCTGGATGCCTTCCGCATT
GCGAAGGAAGCCACCGATGACTTCATTTTGAATGGCCAACTTGGAACATACTACAATGAAATCATGCCAAGATCGACTGAACTCTGTGTTGCCCATATTGTCAATGCTTTTGAACAGCT
CGGATGTCCGATACGCAGTGCAGCAGCGGGTCAAAGACTTGAGCGTGTTCCATACCTTGCAAAACATGAACGATTCATGAACTTGATCTACGGTCTTCTCGAGGATGCCAGATTAATT
GACATCAATGGATCTAAAATCACGAGAACGGCCTTACCTGTTCCGACGAAATCCGTGGAAACAATGCTGGAAGAATTGCTTCGCGACGAGCCTATCCATGCAGCCGAGCACAAACTC
ACATCCATGACCGGAAGTAAGTTTGCCGACTGCATTAGTGGCAAGGAAGACGGGCTTCAATTAATCTTCGGGTCACCCGAGGGCAGAGAAATCGCGACTGACGTCTATGCCAAATCT
CCCATTAATGCGGTATGGATCCAGCAGGCGGAAATGTTCCTCGAGCAGCTTGTGCAAAGGCTACCCAATACTGGTGAGCCTCTGCGCATCTTGGAGATGGGAGCTGGTACAGGAGGA
ACAACTGTGAAGATCCTGCCACTGTTGGAGCGTCTAGGTGTGCCCGTCGAATACACAATGACGGACCTATCATCCTCATTGGTTGCCGCGGCTCGCAAACGATTCAAGAAGTATCCGT
TCATGAAGTTCAAAGTGGTGAACATCGAGTCTCCGCCTGATTCACAACTGGTACATAGTCAGCACATTGTCCTGGCTACAAATTGTGTGCATGCAACTCGGAACCTGGAAGTCTCGAC
GAGAAATATTCACCATATTCTGCGACAAGACGGCTGCCTACTTTTATTGGAGATGACCGAGCAAGTGCCTTGGGTCGATTTCATTTTCGGTTTGCTAGAAGGATGGTGGTTGTTCGAA
GATGATCGGCAACACGCACTCCAACCCGCAACGCACTGGAAGAAAATTCTCACTTCCGTTGGATATGGTCACGTCGATTGGACTGAAGGTGGTCGGCCAGAGGCCAATATCCAACGC
CTGATTATTGCTCTTGCGTCTAAACCCAGGTATGATGATGCACCTAAGCCACTCCAGCCGCCGGCGCATGTCCCATTGACAGAAATCGTAGGGAACCAAGAGATCATTGATACATATAT
CCATGAATATACCAAGGACTTCCACGCACTCCCCATAACGATTACACAGCAAGCCGTGATTCCCGCTCTCACGGGACACTGTGTGCTTGTTACTGGTGCCTCAGGGAGTCTCGGCTGCC
ACATTGTCGGCTATCTCGCCAGGCTTCCTAGTGTCCACACTGTAGTCTGTCTGAATAGACGGACTACGGTGCCCGCTGTCATCCGTCAAGAGGAAGCTTTAAAGGTCAGGGGAATCTC
GCTCGATGACATCTCCCGGTCAAAGCTAGAGGTACTCGAAGTGGAGACCGCAAAACCATTGCTTGGGCTGCCTGTGGAAACCTATCAGAAACTCGTGAACACCGCAACCCATTTAGTT
CACAACGCTTGGCCAATGAGTCTTACCAGACCTATCCGAGTCTATGAGAATCAGTTCAAGGCTATGCGGAACCTCATCACTCTTTCACGGGAGGTAGCTGCACTCCGACCGGCACCATT
CAAGTTCGGCTTCCAATTCATCTCGTCGATCGGGGTAGTCGGCTACTATCCACTTCGCTATGGAAAATCCCTTGTTCCAGAAGAGACAATGACGGCGGATTCTGTCCTATCAGTCGGAT
ATGCTGAAGCAAAGCTTGTTTGCGAGCGCATGTTAGACGAAACGTTGCATCGGTATCCAGATAGATTCCGACCAATGGCAGTAAGGATCGCTCAAATTACTGGCTCAACAAGCAACG
GGCATTGGAATCCAGTCGAGCATTTTTCGTTCCTGATCAAATCCTCGCAGACATTGAAAGCGCTCCCGGACTTTGATGGTAGCCTCTCTTGGTGTCCAGTCGACGATGTTGCCGCAACA

CTGGGCGAATTAATGATTTCTGACACAACGCCTTATTCGATCTACCATATCGAGAATCCATCAAGGCAGCCGTGGCGAAAAATGGTGAAAACACTGGCCCGGTCTCTTGACATCCCAC
AAAGTGGCATTATTCCTTTTGACCAGTGGATTGAACGAGTTCGAAACTCCCCAGCCTCGGTCAACGATTGTCCAGCCAAGCAATTACTAGAATTCTTCGATCAGCATTTTATCCGAATGT
CATGTGGGGGTTTGATACTAGATACGGCTAAGACGAGGGAACATTCAACAACTTTGCGGGAAAGGGGCCCTGTAGGCCCAGGTTTGGTGGATAAGTATATTTTGTCGTGGAAGGCC
GTGGGATTTCTAGATTAA

>g03742_pksCT_citS
ATGGAGGATACAGAGACGTTAGGCCTCTGCACCGGCACCCTGAGTGCTTTAGCAGTGGCATGTTCCTGCAATGTGGCCGATATTCAACACTACGGCGCGGTAGGCGTGAGACTGGCA
ATGTTGGTCGGAGCCACCGTTGATGCTGAAGAGGCGTTATCTGACCCAGAAAGAAAGGCAACGAGTCTTTCAGTGTCATGGAACAGTGCCGAATTCGGTGACTCATTTGCCCATATCC
TCGAGACATTCCCTGATGTATGTATTGACGACTCGTATTCACACCACAGCAAAACACTAATTGAAATCCTTTAG

>g06948_ctnB_citA_serine_hydrolase

ATGGTCGAGACAAATTTCGAGGCAGTCGATGACACTTTACATCTTCCACGAATCCTTTGTTTTCACGGCGGGGGGTCTAATGCTATCATCTTCAAATCCCAGTGCCGCCGGCTTATTGC
CCAACTAAGATCCGAGTTCCGGTTTGTCTTCGCGCAAGGACCATTTTTATCTGACGCTGGGCATGATGTGATGTCGGTGTACAGTCAATGGGGTCCATTCAGGCGTTGGCTCCGTTGG
CGCCCCGACCACCGTGATATCCGACCGGAAGATGCCATCCGAGCGATCGACGACAGTGTGGAGGACGCAAAGCGTCAAGATGACGCCAAAGGAGCTACAGGATTGTGGGTGGGAC
TGCTTGGATTTAGCCAAGGCGCGAAGACCTGCGCTAGTCTCCTCTACCGTCAACAAATCAGACAGGAACTACTCGGACGACCGTTCGCCGGTTCGGACTATCGTTTTGGAGTTCTGTT
GGCTGGCCGTGCGCCGTTAGTATGCTTGGATCTGGATTTAGATATGGGTTCATCTTTGCCTGATGTGTCGCAGATTACGGATGCAAAGTACCACGGTCCGAGCCAGGACGTTCTACGC
ATCCCCACCGTACATGTCCATGGGATGTTAGACCCGCATGTGGATCTTCACCGTCAGCTGTTTGAAGAGTTCTGTGCCCCGGAAAGTAAGAGGCTGGTAGAGTGGGATGGTGATCATC
GGGTTCCGCTGAAGTCCAACGATGTCTCGCTCGTTGCCTATCAGATTCGAGAAATTGCAATGCAGACGAATGCCCACTAG

>g03740_ctnC_MFS_transporter

ATGAAGGATGCAATTGACATCCCAATTTCGACGAACGCTTCTGTCACCGATTTGGAGAATGCCCGAGATCATTCATCTGGCGAGAAGCCCAATGACGCGGATATTCACTTGGTTGAAT
GGGATGGGCCTGATGATCCAGAACTTCCCATGAACTTTCCCTTTTGGAGGAAATCGCTCATCACTTGCATCTTCAGCACATTGACCATCTGGGTGACCTTCTCCAGTAGTGTCTTTAGTG
CTGCGACCACGGTCACTTCCAAAGAGTTTCATGTTTCAAAAGAGGTCATGACTCTAGGAACCAGTTTGACTGTGCTGGGCTTTGCAGTGGGACCACTGATTTGGGGACCCATGTCGGA
GTTATATGGCCGACTAAGGCCTCTCTATATCGGATACGCCATCTTTATCATCTTCCAGGTTCCCGTGGCGGTTGCGCAGAACCTCGAAACCGTGATGCTTGCACGTTTCTTGCTGGGGT
TTTTTGGTACCTCCGCTCTTGCGATCATTCCTGGTGCTCTGGCAGACTTTTGGGGTCCAGTCGAGCGCGCCATTGCCGTCTCTTTATTTTCCGCGGCTACCTTTGTTGGGCCGATATTTG
GACCAATCGTTGGGGGATTTATCGTCGACTCATCTCTAGGATGGCGCTGGACTGCTTGGATCACCATGATCCCAGCCGCATTCTTCGGCACCATCGCTCTCCTCACCCTACCCGAGACA
TACCACCCAGTTCTCCTCCAGCGCCGCGCTAGTCGGCTCCGTAATGAGACACGAATCTGGGCCTACCATTCCCGTCTGGATGAAAGCACGCCGACGTTTAGCCAAATCTTGACGAAAT
ATCTCTTCCGTCCGCTTCATATGCTTTTCTCGGAGCCGATCCTCGTCTGCATGACTCTTTACATCTCGCTTATCTATGGTATATTGTACCTCTTCTTCGTGGCATACCCGATCGCTTTTCGC
GAGGTCCGGGGTTGGACTTCCTTGGGAATTGCGGCCTTGCCTTTCCTGGGCATCCTAGTGGGGGTCCTACTGGGTTGCCTCCTTGTCACCATCGCAACGCGCCTGTGGTATGCTCCTAA
GCTACACAATGGTTCCGTCGTACCGGAAGATCGTCTACCCCCCATGATTGTTGCGGCTTTACTTTTGCCTATCGGGCTATTCTGGTTTGGATGGACATCGAGCCCGAGTATCTCGTGGG
TGCCACAGGCCATTGCAGGAGTGCCAATCGGAATGGGGATTTTGATGATCTGGATGCAAGGCCTGAACTATCTGATTGACGTATATTTGGTGGTTGCCAATTCGGCTCTGTCTGCAAA
CACTCTTATTCGAAGCATGATCGGTGCTGCGTTTCCCCTTTTCGGGTCGGCAATGTACGGTAGGTTAGGAGTGGACTGGGCTATGTCGCTGCTCGGCTTTCTTGCCGTTGCGATGATCC
CGATTCCCATCATCTTCTATTTCTATGGGGCGAAGATTCGGGCCTTGAGTCGCTTCTCTCCAAAGCTGTAA

>g06947_ctnE_shortchain_dehydrogenase

ATGGCTTTTCCACCCTCCGCTGGATTTACTTGGATCTCCAAAATACACAATGACACCTATCCTACCATCACAGCGGCCAGATGTAAGCAGCATGGACGAGCTGTCTTCGTGACCGGTGC
ATCGAAGGGCATCGGCCGCGCGATAGCGATTGCATTCGCTCAAGCCGGTGCCTCCTTCATCGCACTCGGAGCAAGATCCTCCCTCGACGCAGTTGAGACAGCGGTGCTCGACGCTGC
AAAGTCTTCAGGCCATCCACCACCTCATATCCTCAAGGTGGCGCTAGATGTTTCGGATGAGCAGAGTGTGTCCGATGCAGCCGCCGAGGTCAAGCGAGCGTTCAGCTCTCTCGACATC
TTAGTTAACAATGCCGGTCGCGTTGAGAAATGGGTTCCACTTGCGGAGACGGATCCCAAATCTTGGTGGTCGACATGGGAGGTCAATGTAAAGGGCACATACCTCGTGACGAGGGCC
ATGCTGCCCCTGCTTTTGTTAGGGGGCGAAAAGACCATTATCAATATGAACTCCATCGGCGCCCACCTGACTCGGCCCGGTGCCTCGGCCTATCAGACCGGGAAATTAGCGATTTTGC
GCTTGACGCAGTTCACTTCTGTTGAGTACGCTGCTCAGGGAGTTTTGGCCTTTACCATTCACCCGGGCGCCGTGGATACCGAGTTAGCGTCGAGATTGCCCGAGGACACGAAAGCAAA
GCTGGTGGATTCGCCAGAATTAAGCGCCGATACAATTGTCTGGCTAACTCAGGAAAAACAGCTTTGGCTGGCTGGACGCTATTTGAGTGCCACCTGGGATGTGGCAGAGTTGATGGC
TCGGAAGGAGGAAATTGTCCAGGGCGACAAGCTTAAGGTCAAGTTGGTTCTGTAG

>g06944_ctnR_transcriptional factor

ATGCTCTCTCATCAGATGGCTCCCACATCACTTACACATACCTCCCAACCTACCAGACAGCGACAACGAACAGGAAGAGCATGTGAGGAGTGCCGGCGACGCAAGCTACGATGTGAT
GCACTGCAACCGCGGTGCGGGGTCTGTGTGGAAGCAGGTATAACATGCGAAGTCAATAGCCAACGGCAGCCGAGGGGCCCGAAGAAGGGCTACTTGAAGGCATTGAGAAATCGAG
TCGCAATGCTCGAAAATCGTCTACCGACCCAGCATCACCCCGGGCCCTTGCCTGAGCCCAACCCATTGTCAATTCCCTTGAACGACGACCACCATGATGGCTGGAGTGTCTCTAGCGTG
TCGAGCCGTTCCGACTCAAATCCGCCTCCGTTGATCATGGAAAGTACAACCGTCTCCGAGCCAGATGTATCCATATTGAACTCGATGTCGACGTTCGGTTCGGCTCCGTCAGTGGGCAT
GTACGGGAAAGATATCTGTGGGATCGAGCCGATATCAGAGTTCATGCAGGCGGAATTAAATCAACTATATTTTGACCGAGTGCACCCATCCGTCCAGATACTTCACCAGCGTCGTTAT
TTAGGATGGGCCAGAAGCGCCTCAAAGAAGATATCTCGTCGATGTCTGCAGTACGCGGTATGGACACTGGCCTCCCTCCTGTCCGCGCAATTCCAACATCTTCAAGACTCCTTCTACCA
GGAAACAAAGCGGACTTTAGAATTCTCCTATCTATCGGGAGACTCTGATAATTTGGTTGACACAGAAGAGATTCAAGCATGGATTTTGATAGCAACGTATGAGTCAATGCGGACTTTC
CATCGCCCTGCGTGGATGAGTGCCGGACGCGCCTTTCGTCTGGTTCAGTTAATGCGACTGCACGAAATCGACAGTCCTACAAAACCCCCTGTAGCTGATGCAGACCTGATCGAGACAG
AGGAAAAGCGCCGGGTGTTTTGGACGGCCTACTTCTTGGATCACTTATTTAGCATGCGTAATAATTGGCCGATCACCTTGAACGAGCATGTGATCTGCACCCGTCTTCCAGCTCCAGAC
ATGGAATTCCAAAACGGTCAACCGGTGCTAGGTGCCTTCCTTTCGGAAGCGATCATGGACGTTATGCCGCAGACAACATCGCCATTTAACGAATGCGCCATCTTGGCTACCATATGCG
GGCGCAGCCTATTTCAGGCCCAGCAATACAGTGTCCGCTTTGTGTATGGCGACATAGCTCCCAACTGGACCGATCAGCACCAATGGCTGGACAACGTCCTTACCAACCGTCTTCAGAT
CCTTTCCCAACACTACCCCTCGCCAACTCAAGTCTGCGACCCGATGCTCTCGTTCGCACACATTATGGGACAGGCAAGTGTGATTCATCTATACAAGGGCATGGAATCGATTGTGTGGG
CAGTCGACGAGGGAGCATTAGTATTAGAATATCGGCGGCGTGCCCTAAGTGCAGCGCAGGAGATCGTCAAGCAAGCAAAAGGACTGATAGAGTTCTACTTCTTCAAGGTATGCCACA
CTGCATTACTACTTCAGTCGACTTGGGACATGAATATCTTTACGCACCGCGAACGTACTAATTATAAAACGAGCAGGTCCATCCTCTCATGCCCATCCCATTATTACTCTGCGCCGAATT
CCTCTACAGTAATCGAGGGTCAGACGCGGCATTTAACTCGCTACTTCAAGAGCTTCTACAGATTTTTCGTCAACTCAAGAATCCTAACGATCCAACTCAAAGCTATATACATCTTCTAG

>g06943_ctnD_citC_oxidoreductase

ATGTCTCAAAAATTGGAAATCATGGCCACAGACAAGCTTGTCGATTTTATCCAGACACCCTTTGACTTTCTCATCGTCGGCGGCGGAACTGCCGGTCTCGTCCTCGCAGCTCGTCTTTCT
GAGGAACCAAGCATTCAAGTCGGGGTAATCGAAGCTGGCTCCCTTAGGCTGGGGGATCCCAAGGTCGACCTTCCCACAGGACCGGGCCAGATGATAAGCAATCCTGACTATGACTG
GAACTTTGAGAGCATCCCACAGGCTGGCACCAATGGCAAATCCTATCATATTCCTCGAGGAAAAATGCTAGGTGGCTCGAGCGGCATCAACTTCATGTCTTATAACCGGCCATCTGCC
GAAGACATTGATGATTGGGCCAATAAGCTTGGTGTCACAGGGTGGACATGGTCTGAACTACTACCGTACTTCAAAATAAGTGAAGGCTTGGAGCCCGTCAAGCCCAGTACAAGTTGT
CCAGTGGAGCCCAAGGTCCATGGCACCGATGGGCCAATACACACTTCGATAGGCCCCTGGCAACCGCTAATAGAAGAATCAATTTTGGCTGCGTTTGATGAAACCTCTCGCCTTCAGC
GGCCAGTCGAGCCCTACGATGGTACTCATTTGGGCTTCTACAGGTCCTTGTTTACACTGGACAGAACCAGCAAACCAGTTCGAAGCTATGCGGCCAGCGGTTATCTTGCTCCCATTATG
GGTCGCCAAAATCTGAAAATCCTTGAAAACGCACAGGTGTGCCGCATCCTACTCTCCGATGCTCCAGATGGGACACCCACTGCAGAAGGGATTGAATTGCAGCACATGGAGGCTCGC
TACACCGTCTCAACTAAAAGAGAAGTCATACTCAGCGCCGGGTCTATCCAGAGTCCCCAGCTTCTAGAGCTTTCTGGAGTTGGAGACCCTAGTGTCCTCGAAAGCGCTGGGATTGCTT
GCAGAGTGGCTATCACGGACGTGGGCAATAATCTACAAGAACACACGATGTCAGCTGTATCTTATGAGCTCGCGGATGAAATTATATCGGTGGACTCCTTATTCAAAGATCCTGCCTT
GCTACAAGAGCATCAAAGGCTCTACGCCGAAAACCATTCCGGAGCCTTGTCTGGATCGGTTAGTCTAATGGGCTTTACTCAATACTCATTACAATCCACCGAAACCCAAGTGAACGAC
ACTGTGGCGCGTATCTTCGACGCCCCCAGTCTGGGCGGCGAACGGTTTCAGCAAAACGCCAGTTACCAGCGCAAGCAACAGGAGGCTATTGCCGGCCGGATGCAAAATTTCCAGTCC
GCGGATATCCAGTTCATTGGCACCCCCGCGTATTTCAACACCACTGCCGGGTACAGAAACTGCGCCAAGATCGCATCTGGCTCTCCTGTCGGCTACAACGCATGCTACTCCATCGTCGT
CAGCAACATGTATCCTTTATCTCGCGGGAGTGTACATGTGCGGACCTCGAATCCAATACAGGCACCGGAGATCGATCCGGGATTTCTTCGTCATCCCGTGGATGTTGACGTTCTTTCCT
CTGGTATTGTATTCGCAGACCAAGTGTTCCAGTCGACCTCCCTAATCGGCAAAATTGGTCGGCGAGTGAGCCCCCCTGCTAAGCTCAATCTGTCGAATATGGATGAAGCTCGCCAATTT
GTTCGCAATCACATCGTGTCCTTCGTGTTGTTGATGCCAGTGTCATGCCGATGCAGGTCAGTGCCGCGATCATGGCTACTGTGTATGCCATTGCAGAAAAAGCGTCGGATATTATCAA
GGAGGACGCTGGGTTTGGCACTCGGTCAAGTGCTCGTCTGTGAGCTGGATTTTGTAGATATATTTATAATAGTTCACGTCTCATGCTTATTCCGATCTTTTCCGCTCCAAATCGAGTACC
TCATTGCAGCTGCATTCATATTATAA
